# Free indirect discourse as logophoric context

**DOI:** 10.1007/s10988-025-09441-z

**Published:** 2025-11-20

**Authors:** Isabelle Charnavel

**Affiliations:** Linguistics Department, University of Geneva, Geneva, Switzerland

**Keywords:** Free indirect discourse, Logophoricity, Attitude, Indexicality

## Abstract

This article argues for a logophoric analysis of Free Indirect Discourse (FID). FID is descriptively a hybrid between Direct Discourse (DD) and Indirect Discourse (ID). Recent studies largely agree on a DD-based analysis of FID by relying on bicontextual dependency (Schlenker in Mind Lang 19:279–304, 2004, Eckardt, The semantics of free indirect discourse: How texts allow us to mind-read and eavesdrop, Brill, Wiley, Leiden, 2014, i.a.) or mixed quotation (Maier in Mind Lang 30:345-373, 2015, i.a.). Instead, the article defends an ID-based, logophoric analysis of FID on the basis of overlooked properties of FID such as (anti)licensing of (anti)logophoric elements and recursive embedding of FID, which strengthen some previously discussed arguments such as de se and de te readings or sequence of tense phenomena (see Sharvit in Linguist Philos 31:353–395, 2008); the new observation that time and location adverbials are in fact not systematically indexicals anchored to the protagonist (but can be anaphoric or anchored to the speaker) further supports ID-based against DD-based analyses. The hypothesis that FID is outscoped by a logophoric operator not only derives the mixed properties of FID, but also treats FID as a case of an independently motivated linguistic class—the class of logophoric contexts.

The goal of this article is to defend the hypothesis that Free Indirect Discourse (FID) belongs to the class of logophoric contexts.

Descriptively, FID, which is illustrated in French in (1), appears to be a hybrid between Direct Discourse (DD) and Indirect Discourse (ID).

**Table:** 

(1)	Que faire ? … C’était dans vingt-quatre heures; demain! Lheureux, pensa-t-elle, voulait sans doute l’effrayer encore. [Flaubert, *Madame Bovary*]
	‘What was there to do? It was in twenty-four hours; tomorrow! Lheureux probably wanted to scare her again, she thought.’ (cf. Banfield, 1982, p. 98)

On the one hand, the discourse in (1) exhibits some properties of a DD (inwardly) uttered by Emma Bovary: it contains questions and exclamations representing Emma’s thoughts, as well as an indexical (*demain* ‘tomorrow’) anchored to Emma’s temporality. On the other hand, Emma is referred to by a third person pronoun and the discourse is narrated in the past, which are characteristic of ID.

For these reasons, FID is often considered in the literary tradition as a style presenting a character’s consciousness through the narrator’s voice. Although it is attested much earlier in literature (already in the Middle Ages—see, e.g., Cerquiglini, 1981—or even Antiquity—see, e.g., Biraud & Mellet, 2000), FID was first theorized only at the end of the 19th century (especially in French as *style indirect libre*^1^)*.* From a formal linguistics’ perspective, the study of FID was pioneered by Banfield (1982). The observation that FID is not confined to literary texts, but appears in everyday language, as exemplified in (2), may have then favored the development of linguistic analyses in the past 40 years (see Doron, 1991; Schlenker, 2004; Sharvit, 2008; Eckardt, 2014; Maier, 2015, i.a.; see review in Reboul et al., 2016).

**Table:** 

(2)	X a appelé. *Est-ce qu’on peut l’emmener, toi ou moi, à Roissy, à 5 heures du matin, demain ?* Il rêve !
	[oral 10–04–2005] (Authier-Revuz, 2020, p. 314)
	‘X called. *Can we drive him, you or me, to Roissy, at 5am tomorrow?* He is dreaming!’^2^

Recent debates about FID center on two main types of analyses. As we will detail in Sect. 3, the first one, proposed by Schlenker (2004) and extended by Eckardt (2014), relies on dual-context dependency: under this hypothesis, the mixture between DD and ID properties in FID is due to the fact that both the protagonist’s and the narrator’s contexts are accessible in FID, and expressions are relativized to one or the other depending on their class (as in Schlenker, 2004) or their lexical entries (as in Eckardt, 2014). The second one, defended by Maier (2015, 2017), treats FID as a case of mixed quotation, some parts of which can be pragmatically unquoted. Both types of analyses thus make FID closer to DD than ID, in the sense that the protagonist’s perspective is rendered in FID by direct access to her context without any mediation to the narrator’s context.

Instead, the present article defends an analysis of FID that makes it closer to ID, in the spirit of Doron (1991) and Sharvit (2008). The core idea consists in positing the presence of a syntactically represented logophoric operator (see, e.g., Charnavel, 2019a, 2020) that semantically embeds FID. Logophoric operators, which have been posited for reasons independent of FID, are similar to implicit underspecified attitude verbs imposing the perspective of their subject in their scope, thus forcing *de se* and *de dicto* readings and (anti)licensing (anti)logophoric elements. This hypothesis—henceforth the log-FID hypothesis—will not only be motivated by the peculiar perspectival properties of FID already observed in previous literature, but also by new arguments, such as recursive embedding of FID, behavior of reflexives and ban on anti-attitudinal elements—facts that have been either overlooked or left unexplained in previous analyses.

Section 1 starts by laying out the empirical arguments for an ID-based, logophoric hypothesis against the class of DD-based analyses. Section 2 presents the specific ID-based analysis defended here, i.e., the log-FID analysis, as well as some predictions and future prospects. Section 3 details how previous implementations of DD-based analyses are challenged by some (newly observed) empirical properties of FID.

In order to understand the linguistic (vs. stylistic) constraints on FID, note that I will use a variety of data in different languages (French, English, German, Hebrew, Italian) ranging from literary to constructed examples, including some from linguistic articles and oral corpora. Further note that I will mainly restrict myself to FID representing speech and thought, and exclude from the analysis FID representing pre-linguistic perception, or so-called Protagonist Projection (PP) illustrated in (3) (see, e.g., Abrusán, 2021); we will nevertheless briefly discuss in Sect. 2.4 the prospects of extending the log-FID analysis to PP.

**Table:** 

(3)	The train was full of fellows: *a long long chocolate train with cream facings... The telegraph poles were passing, passing*. [Joyce, *Ulysses*] (Abrusán, 2021, p. 839)

## Empirical arguments for FID as a case of logophoric context

This section lays out the empirical arguments for the hypothesis that FID is a logophoric context. As previewed in Table 1, not only are the arguments against attitude embedding (described in Sect. 1.1) not convincing (as shown in Sect. 1.3), but there are also (old and new) empirical arguments strongly supporting logophoric embedding (described in Sect. 1.2).

**Table 1:** Preview of empirical arguments distinguishing between ID- and DD-based analyses (✓: fact directly explained; ?: fact not straightforwardly explained)

	ID-based analyses	DD-based analyses
Root clause syntax	?	✓
*De re non *de dicto* readings	?	✓
Evaluative expressions anchored to protagonist (epithets, expressives, speaker-oriented adverbs, discourse particles)	?	✓
Protagonist’s manner of speaking	?	✓
Adverbial indexicals	?	✓
Third person pronouns: person number/gender *de se/de te*	✓ ✓ ✓	? ? ?
Tenses/mood: logophoric (SOT, *Konjunktiv*)	✓	?
Logophoric reflexives	✓	?
*Anti-attitudinal elements	✓	?
Recursive FID	✓	?
Person indexicals	✓	?
Anaphoric adverbials	✓	?
Narrator’s style	✓	?
*Vocatives (and imperatives)	✓	?

As we will see in Sect. 2, the log-FID analysis can derive both ID properties (esp. pronouns and tense) and DD properties (esp. protagonist’s perspective), as well as specific (anti)logophoric properties (e.g., exempt reflexives) because it involves an independently motivated operator (akin to an implicit underspecified attitude verb) that imposes the perspective of its subject in its scope. More specifically, the log-FID analysis likens FID to both ID—because it involves an operator resembling attitude verbs, thus deriving the ID-like distribution of tense and pronouns—and to DD—because this intensional operator forces the anchoring of perspectival elements in its scope to its subject. By contrast, what I call DD-based analyses (namely, bicontextual and mixed quotation accounts that both make FID closer to DD than ID) straightforwardly derive DD properties, but are challenged by ID properties. Although I here conflate bicontextual and mixed quotation accounts for simplicity, note though that their predictions about ID properties differ in some places, as will be detailed in Sect. 3.

### Similarities to DD

#### Root clause syntax

In FID, the narrator (or speaker) reports the perspective of a protagonist (or any other contextually salient and relevant individual). In that respect, FID resembles ID. But the main reason why it is agreed upon that FID differs from ID is that FID does not exhibit the syntactic complementation properties of ID, as already shown in detail by Banfield (1982).

First, FID is incompatible with a complementizer (hence the traditional label *Free* Indirect Discourse). An attitude verb can optionally appear in FID as a parenthetical as in (1) (*pensa-t-elle* ‘she thought’), but it cannot introduce FID with a complementizer as in standard attitude reports like (4) (contrasting with (1)).

**Table:** 

(4)	Elle pensa que Lheureux voulait sans doute l’effrayer encore.
	‘She thought that Lheureux probably wanted to scare her again.’

This is corroborated by the fact that FID does not involve the syntax of embedded clauses: in FID, we routinely observe inverted questions, exclamations, repetitions, hesitations or incomplete clauses, which are characteristic of root clauses.

**Table:** 

(5)	*Mais s’il y avait quelque part un être fort et beau, une nature valeureuse […], pourquoi, par hasard, ne le trouverait-elle pas ? * *Oh ! quelle impossibilité !*
	[Flaubert, *Madame Bovary*]
	‘*But if there had been somewhere a strong and handsome individual, a gallant nature […], why, by* *chance, should she not find him? Oh, what an impossibility!*’
	(Banfield, 1982, p. 74)
(6)	His wife still loved him, physically. But, but—he was almost the unnecessary party in the affair.
	[Lawrence, *England, My England*] (Banfield, 1982, pp. 74–75)

For example, the question and exclamations in (5), as well as the repetition in (6), are all unacceptable when embedded under appropriate attitude verbs:

**Table:** 

(7)	a.	*Elle se demanda pourquoi, par hasard, ne le trouverait-elle pas.
		‘*She wondered why, by chance, should she not find him.’
	b.	*Elle s’exclama oh quelle impossibilité.
		‘*She exclaimed oh, what an impossibility.’
	c.	*He thought that but, but—he was almost the unnecessary party in the affair.

It is thus clear that FID does not involve subordination and syntactically behaves like DD rather than ID.

#### Protagonist’s privileged perspective

For many, this syntactic resemblance to DD (rather than ID) is corroborated by a semantic resemblance: to the exception of tenses and pronouns (see details in Sect. 1.2.1), FID exclusively expresses the protagonist’s, rather than the narrator’s perspective.

First, *de re* non *de dicto* readings, which are acceptable in ID, are prohibited in FID (Reinhart, 1975, 1983; Schlenker, 2004; Sharvit, 2008, i.a.); for example, the definite description *his mother* in (8) can be interpreted *de re* only in (8)a, rendering contradictory Oedipus’s thought expressed in (8)b.

**Table:** 

(8)	a.	Oedipus believed that his mother was not his mother.	(Reinhart, 1975, p. 132)
	b.	#*His mother was not his mother,* Oedipus believed.	

Second, all evaluative expressions—including epithets (e.g., French *ce grand fat* ‘the conceited ass’ in (9)), expressives (e.g., French *cette damnée crise* ‘this damned crisis’ in (10)), speaker-oriented adverbs (e.g., French *franchement* ‘frankly’ in (11)), discourse particles (e.g., German *wohl* in (12) expressing that the speaker has imperfect evidence for the truth of her utterance, see Eckardt, 2014)—must be interpreted from the protagonist’s point of view in FID, just like in DD (Banfield, 1982; Eckardt, 2014; Maier, 2015; Schlenker, 2004; Sharvit, 2008, i.a.).

**Table:** 

(9)	Ma tante continuait ; mais je n’écoutais plus ; une seule chose m’importait : *Alissa refusait de se marier avant sa sœur. – Mais Abel n’était-il pas là ! il avait donc raison, ce grand fat*.
	‘My aunt kept talking; but I was not listening anymore; one thing alone mattered to me: *Alissa refused to marry before her sister. — But Abel wasn’t there! He was then right, the conceited ass!*’
	[Gide, *La Porte Étroite*] (cf. Banfield, 1982, p. 73)
(10)	*On devait*, disait-il, *trouver là de l’or à la pelle. L’idée était juste. Seulement, le million y avait passé et cette damnée crise allait lui donner raison.*
	[Zola, *Germinal*]
	‘*One ought*, he said, *to find gold in one’s shovel down there. The idea was good, only he had already sunk a million in it and this damned crisis was going to prove him right*.’
	(Banfield, 1982, p. 90)
(11)	Pour savoir où s’établir, ils passèrent en revue toutes les provinces. *Le Nord était fertile, mais trop froid . . . et le Centre, franchement, n’avait rien de curieux*.
	[Flaubert, *Bouvard et Pécuchet*]
	‘In order to know where to set themselves up, they considered one by one all the provinces. *The North was fertile, but too cold . . . and the Center, frankly, had nothing of interest*.’
	(Banfield, 1982, pp. 117–118)
(12)	(…) Und er ging in ein kleines Wirtshaus, ließ sich eine Speise auftragen und trank Wein dazu. Er aß langsam; er wartete von Bissen zu Bissen. *Über der Eingangstür war eine Uhr … sie war wohl stehengeblieben …*
	[Schnitzler, *Reichtum*] (Eckardt, 2014, p. 132)
	‘… and he [= Weldein] went into a small inn, let them serve him a dish, and drank wine with it. He ate slowly; he waited from bite to bite. *Above the door was a clock … it had wohl stopped*.’

Third, FID can reflect the manner of speaking of the protagonist, at least to some degree (Banfield, 1982; Maier, 2015; Schlenker, 2004, i.a.). For instance, the character Camus’ nonstandard dialect (whose main lexical and syntactic characteristics are underlined) is used in the FID in (13), while the rest of the narration uses standard French language.^3^

**Table:** 

(13)	Faudrait peut-être pas aller ce soir, hasarda Lebrac pensif. Camus bondit — *Pas aller ! Ben il la baillait belle le général. Pour qui qu’on le prenait, lui, Camus ! Par exemple, qu’on allait passer pour des couillons* ! Lebrac ébranlé se rendit à ces raisons. [L. Pergaud, *La guerre des boutons*]
	‘We shouldn’t go tonight, Lebrac ventured, thoughtful. Camus jumped up. *Shouldn’t go! Well, how did the general dare spin them a line! Who did he think he – Camus – was! They would be thought to be bloody idiots*! Lebrac, shaken, listened to such reason.’
	(Authier-Revuz, 2020, p. 317)

Last but not least, adverbial indexicals in FID like French *demain* ‘tomorrow’ in (1) or *hier/avant-hier/aujourd’hui* ‘yesterday/the day before yesterday/today’ in (14) are interpreted relative to the protagonist’s temporality (Banfield, 1982; Doron, 1991; Eckardt, 2014; Maier, 2015; Schlenker, 2004; Sharvit, 2008, i.a.). This property is usually considered to be a hallmark of DD.

**Table:** 

(14)	Descendu de cheval, il allait […] et souriait, étrange et princier, sûr d’une victoire. *A deux reprises, hier et avant-hier, il avait été lâche, il n’avait pas osé. Aujourd’hui, en ce premier jour de mai, il oserait et elle l’aimerait*.
	[Cohen, *Belle du Seigneur*]
	‘Down his horse, he was walking […] and he was smiling, strangely and princely, certain of a victory. *Twice, yesterday and the day before yesterday, he had been a coward, he hadn’t dared. Today, on this first-of-May day, he would dare and she would love him*.’ (Authier-Revuz, 2020, p. 135)

In many respects, FID thus patterns with DD, which motivates many analyses under which FID is directly anchored to the protagonist’s context, whether it is through quotation (Deal, 2020; Maier, 2015, 2017, i.a.) or bicontextual dependency (Eckardt, 2014; Schlenker, 2004, i.a.), as we will see in more details in Sect. 3.

In the rest of this section, I will instead present empirical arguments that FID is embedded under some kind of attitude operator (cf. Doron, 1991; Sharvit, 2008): several additional properties of FID, which are often overlooked, demonstrate that FID has to be (semantically) embedded. Furthermore, the properties we have just surveyed do not necessarily argue against the (semantic) embedding of FID.

### Similarities to (logophoric) ID

#### Pronouns and tenses

As has long been noticed, personal pronouns and tenses in FID correspond to those in ID (see Banfield, 1982; Schlenker, 2004; Sharvit, 2008; Eckardt, 2014; Maier, 2015, i.a.). For example in (14), the protagonist (Solal) is referred to by a third person pronoun, even in the FID passage characterized by the DD properties described above. Similarly, the FID passage uses past tense, which is anchored to the narrator’s (vs. Solal’s) temporality. These two properties are found in the ID counterpart of (14) in (15).

**Table:** 

(15)	Solal se disait qu’à deux reprises, […] il avait été lâche.
	‘Solal was telling himself that twice, […] he had been a coward.’

As detailed in Sect. 3, these properties impose an explanatory burden on analyses that liken FID to DD: to account for them, quotation theories assume unquotation (Maier, 2015, i.a.); bicontextual theories assign a specific contextual anchoring to some (presuppositional) expressions (Eckardt, 2014; Schlenker, 2004, i.a.). On the contrary, these properties straightforwardly follow from an account likening FID to ID.

Furthermore, the analysis of pronouns and tenses in FID must take into account three types of complications, whose exact empirical properties remain debated: the interpretation of gender and number features, the distribution of first and second person pronouns, and the absence of non *de se* (and *de te*) pronouns.

First, it seems that the gender feature of a third person pronoun referring to the FID protagonist reflects the narrator’s judgment (see (16)a), while the gender features of pronouns referring to other individuals reflect the protagonist’s judgment (see (16)b)^4^ (see Doron, 1991; Schlenker, 2004; Sharvit, 2008).

**Table:** 

(16)	a.	[In her dream, Mary_i_ was a cardinal.]
		*Really*, she_i_ thought, {*she*_*i*_*/#he*_*i*_} *had excellent chances of becoming Pope some day*.
	b.	[Mary wrongly believed that Robin was male. In fact, Robin was a woman.]
		*Where was he this morning, for instance*? (Mary wondered)
		(Schlenker, 2004, pp. 290–291)

Thus, Mary in (16)a, who is the center of thought, must be denoted by a feminine pronoun, even if she wrongly believes herself to be a man in the dream. But in (16)b, the pronoun designating Robin must reflect Mary’s (wrong) judgment (see also Sharvit, 2008, p. 370). This contrast (between cases where mistaken gender concerns the protagonist herself and cases where it concerns a third party) challenges theories that liken FID to DD:^5^ as detailed in Sect. 3, they must stipulate the difference.

These facts, however, follow from an analysis likening FID to ID, as shown in (17).

**Table:** 

(17)	a.	Mary_i_ dreamed (that she_i_ was a cardinal and) that {she_i_/#he_i_} had excellent chances of becoming Pope some day.
	b.	Mary_i_ (wrongly believed that Robin_k_ was male, and) wondered where he_k_ was.

Some variety in judgments is observed for sentences like (17)b (partly depending on whether the content of the parenthesis is included or not), but crucially, the following contrast as a whole holds robustly: while the protagonist herself (Mary in (17)a) must be referred to by a pronoun expressing her actual gender, other characters can be referred to by pronouns reflecting the protagonist’s wrong judgment; as in the case of evaluative expressions reviewed in Sect. 1.1.2, the difference between FID and ID in this latter respect is that gender features must be read *de dicto* in FID vs. ID.

As noted by Abrusán (2021, p. 848) and Sharvit (2008, p. 393), the same holds of number features, which must reflect the protagonist’s judgment, which may be incorrect, as in (18):^6^

**Table:** 

(18)	She was so drunk Fred looked like two guys to her. *Oh no! Now they were coming towards her*!
	(Abrusán, 2021, p. 848, attributed to Stokke p.c.)

Second, the distribution of first and second person pronouns is subject to some constraints that remain debated. According to Banfield (1982, p. 132, cf. Doron, 1991; Cumming & Sharvit 2016, fn. 10), a first person pronoun can appear in FID under two conditions: either the narrator is the same person as the FID protagonist (often a past self, as in (19), where the interpretation of the indexical *maintenant* ‘now’ supports the FID interpretation of the passage), or in cases of represented speech, the narrator is the protagonist’s interlocutor at the time of the narrated event (as made clear by the parenthetical in (20)).

**Table:** 

(19)	*Les désirs qui tout à l’heure m’entouraient, d’aller à Guermantes, de voyager, d’être heureux, j’étais maintenant tellement en dehors d’eux que leur accomplissement ne m’eût fait aucun plaisir. Comme j’aurais donné tout cela pour pouvoir pleurer toute la nuit dans les bras de maman !*
	‘*The longings by which I had just now been absorbed, to go to Guermantes, to travel, to live a life of happiness — I was now so remote from them that their fulfilment would have afforded me no pleasure. How readily would I have sacrificed them all, just to be able to cry, all night long, in the arms of Mamma*!’
	[Proust, *Du Côté de chez Swann*] (Banfield, 1982, pp. 94–95)
(20)	*Did I really know the road?* Ralph asked me. *Were the muleteers to be trusted?*
	[Brenan, *South from Granada*] (Banfield, 1982, p. 123)

According to Schlenker (2004) (cf. Sharvit, 2008, p. 358), Banfield’s conditions are too strict: the first person is also acceptable if the narrator is present (without being the addressee) at the time of the reported speech, as illustrated in (21), or if the narrator has (at the time of the narrated event) magical powers that allow her to read other people’s minds as shown in (22).^7^

**Table:** 

(21)	a.	*Oh how extraordinarily nice I was*, she told my father, without realizing that I was listening to their conversation.
	b.	[Reporting on thoughts I read in my mother’s diary]. *Oh how extraordinarily nice I was*, she thought. (Schlenker, 2004, p. 290)
(22)		I had become adept at reading my teachers’ thoughts. My Greek teacher didn’t like me so much after all. *Really, I was a little devil, not entirely without talent, but impossible to deal with — something had to be done about me, or else tomorrow I would become completely unbearable*.
		(Schlenker, 2004, p. 290)

Under theories based on quotation (Maier, 2015) or bicontextual dependency (Schlenker, 2004), the interpretation of first person pronouns should follow the same rules as that of third person pronouns; in particular, their person features are the narrator’s responsibility. The further constraints on their distribution must thus come from an additional principle, which Schlenker (2004, pp. 289–290) attributes to the pragmatics of narration: if the narrator presents herself as taking part in the action she is reporting, there must be a plausible reason for her to have information about the represented speech or thoughts at the time of the narrated event. We will come back to the issue below in Sect. 1.2.3 (esp. regarding the plausibility of (22)), but it is worth noting already that analyses likening FID to ID can straightforwardly account for the acceptability of the first person in FID: first person pronouns are all perfectly natural in the ID counterparts of (19)-(22), as shown in (23)-(26).^8^

**Table:** 

(23)		Je pensais que j’aurais bien donné tout cela pour pouvoir pleurer toute la nuit dans les bras de maman.
		‘I thought that I would readily have sacrificed them all, just to be able to cry, all night long, in the arms of Mamma.’
(24)		Ralph asked me if I really knew the road.
(25)	a.	She told my father how extraordinarily nice I was, without realizing that I was listening to their conversation.
	b.	My mother wrote in her diary how extraordinarily nice I was.
(26)		My Greek teacher thought that I was a little devil.

Although the same considerations are expected to apply to second person pronouns, they usually do not receive the same treatment as first person pronouns under the aforementioned theories. While most analyses (Doron, 1991; Maier, 2015; Schlenker, 2004; Sharvit, 2008) do not discuss the use of second person pronouns in FID, Banfield (1982, pp. 119–121) explicitly claims that second person pronouns are excluded from FID; according to her, this derives from the fact that FID appears in narration, not in communication. But in fact, we do observe cases of FID in communicative contexts including an addressee to the narrator, who can thus be referred to by a second person pronoun. As illustrated in (27), the narrator’s addressee can correspond to the FID protagonist (cf. Eckardt, 2014, p. 51).

**Table:** 

(27)	[Après le succès à un concours] Rappelle-toi… *tu n’étais pas au niveau ! Tu avais des lacunes fatales*, c’était ton mot, *les autres étaient bien mieux préparés*… Tu avais presque réussi à nous faire douter…
	[oral, 06–02–2011] (Authier-Revuz, 2020, p. 130)
	‘[After the success at a competition] Remember, *you were not good enough, you had fatal weaknesses* – it was your word – *the other people were better prepared*… You almost managed to make us doubt…’

Here, the FID is included in a conversation (from an oral corpus) between a speaker and an addressee after the latter succeeded in a competition; the speaker reminds the addressee how (s)he had worried (s)he was not good enough, using FID to represent her past worries.

Furthermore, a second person pronoun can also appear in FID, even if the narrator’s addressee does not correspond to the protagonist, but to a participant in the narrated event, as exemplified in (2) and (28).

**Table:** 

(28)	Nous nous inquiétions tous sur ton sort. *Qu’allais-tu devenir, après cela?*
	‘We were all worried about you. *What would you become after this?*’

In (2), the present tense of the FID makes the addressee directly salient in the narrated event as she is present at the other end of the line together with the speaker.^9^ In (28), the speaker relates some past event involving both herself and her addressee. The FID represents past worries of the narrator and her entourage about the addressee’s fate at that time, who may have addressed her directly (thus saying *you*) or talked about her in her absence (thus saying *she*). From the addressee’s involvement in the narrated event, we imply some (e.g., family or friendship) relationship between the narrator and the addressee spanning at least from the time of the event to the time of the utterance.

These examples thus demonstrate that Banfield’s assumed ban on second person pronouns in FID is incorrect. As long as the narrator’s addressee is involved in the reported situation, (s)he can be referred to by a second person pronoun. This fact must be explained under approaches assimilating FID to DD, in which *you* would be transposed from a first or third person pronoun. Under an approach likening FID to ID, however, this fact directly follows as the ID counterparts of (27)-(28) or (2) all naturally contain a second person pronoun:

**Table:** 

(29)	a.	You thought that you were not good enough.
	b.	We were all worried about what you would become.
	c.	He has asked whether we—you or me—can drive him to Roissy at 5am tomorrow.

Expressions implying an addressee, which are as little discussed as second person pronouns, seem to raise further challenges to theories of FID as a kind of DD. First, the few studies that mention vocatives (Banfield, 1982; Eckardt, 2014) agree on the fact that they are prohibited from FID as illustrated in (30)–(31).

**Table:** 

(30)	No, (*sir,) he could not obey his orders, he told the officer.	(Banfield, 1982, p. 114)
(31)	The teacher turned to Tom. This, {*his/*my son}, was his worst essay.	(Eckardt, 2014, p. 230)

In both cases, Banfield (1982) and Eckardt (2014) consider cases where FID would report a discourse including a vocative, and observe that the vocative, just like a second person pronoun, cannot be shifted to the reported context. Furthermore, variations on (27), (28) and (2) in (32) show that vocatives intended to address the actual addressee are also banned from FID.

**Table:** 

(32)	a.	Remember, *you were not good enough, (#my son,) you had fatal weaknesses*.
	b.	We were all worried about you. *(#Honey,) what would you become, after this*?
	c.	X has called. *Can we drive him, you (#my dear) or me, to Roissy at 5am tomorrow*?

These facts straightforwardly derive from a theory likening FID to ID, where vocatives are also banned under both interpretations as shown in (33). Under theories likening FID to DD, the absence of shifted interpretations is puzzling.

**Table:** 

(33)	a.	The teacher told Tom that this, {*his/*my} son, was his worst essay.
	b.	We all worried about what (#honey,) you would become after this.

The facts about imperatives are more controversial. Banfield claims that they are banned from FID (as shown in (34)) unless they are not intended to actually involve the reported addressee (as in (35)).

**Table:** 

(34)	*Fix his_*i*_* dinner*, he_i_ ordered. (Banfield, 1982, p. 113)
(35)	*He was really*, Lily Briscoe thought, *in spite of his eyes, but then look at his nose, look at his hands, the most uncharming human being she had ever met*.
	[Woolf, *To the Lighthouse*] (Banfield, 1982, p. 113)

Sharvit (2008), on the contrary, claims that imperatives are allowed in FID and provides as support for her claim example (36), where the narrator, a woman whose husband has been murdered, describes her last phone conversation with him to the police.

**Table:** 

(36)	*Go ahead, start dinner without him* (, he said). [from TV series *Law and Order*] (Sharvit, 2008, p. 368)

Maier (2015) also assumes—without exemplifying his claim—that FID can contain imperatives. Eckardt (2014), however, contends that imperatives are prohibited in FID as evidenced by (37).

**Table:** 

(37)	Der Lehrer sprach Tom an. *Komme/käme zu ihm (sagte er).
	‘The teacher talked to Tom. *Come to him (he said).’ (Eckardt, 2014, p. 229)

To my knowledge, no study considers an imperative involving the actual addressee (participating in the narrated event), but (38) suggests that it is also impossible in FID.

**Table:** 

(38)	I was worried about you and tried to make you react. (#*Hurry up now or) you would never reach your goal!*

These facts are surprising under an DD-based analysis of FID, which should predict that imperatives can routinely appear in FID (see further discussion in Sect. 3). But they seem to be derivable if FID is treated as a kind of ID. Interestingly, the facts about embedded imperatives in ID within and across languages are also controversial (see, e.g., Kaufmann, 2012). In English, Crnič and Trinh (2009) show that imperatives can be embedded only under *say*; strikingly, the contrast between (34) and (36) follows this pattern. A full investigation of imperatives in FID is beyond the scope of the article, but these observations at least suggest that imperatives in FID pattern like in ID rather than DD.

The last class of elements involving the addressee is the class of addressee-oriented adverbs and particles. Banfield includes in the former category adverbs like *confidentially*, *honestly* or *frankly*, as well as expressions like *between you and me*, and claims that only *frankly* can be found in FID (see (11)), which she thus treats as a speaker-oriented adverb; for her, addressee-oriented expressions are banned from FID.

**Table:** 

(39)	{#Confidentially/*Between him and her}, *how extraordinarily nice workmen were*!
	(Banfield, 1982, p. 117)

Eckardt (2014) challenges Banfield’s claim by showing that particles with addressee-oriented content such as German *ja* (conveying that the speaker believes that the addressee might already know the content of the sentence) or English *of course* (signaling that the asserted content should be already known to the addressee) can occur in FID.

**Table:** 

(40)	Mrs. Bartleby mounted the train. *She had made a reservation, of course*.
	(Eckardt, 2014, p. 114)

This variety of facts again seems to support an ID-based vs. a DD-based analysis of FID: while all addressee-oriented expressions can occur in DD, only some are compatible with ID.^10^

**Table:** 

(41)	a.	He said that (*between him and her,) workmen were extraordinarily nice.
	b.	Mrs. Bartleby said that of course, she had made a reservation.

In sum, the distribution of first and second person pronouns, as well as expressions whose meaning involves the speaker and the addressee, seems to mostly pattern like in ID, but rather differently from DD. This supports an analysis treating FID as embedded discourse.

The third complication regarding pronouns in FID is the issue of (non) *de se* pronouns (see Schlenker, 2004; Sharvit, 2008). Schlenker (2004) explains how the *de se* reading of *his* in (42) does not straightforwardly follow from theories that treat pronouns as directly depending on the utterance context (through bicontextual dependency or unquotation), as is the case of DD-based theories, which must account for the non-DD distribution of pronouns.

**Table:** 

(42)	*His*_*i*_* pants were on fire*, John_i_ thought. (Schlenker, 2004, p. 294)

Under such theories, the reference of *his* simply corresponds to the narrator’s referential intentions, without taking into account the content of the protagonist’s thoughts (i.e., whether John had a first person thought or—due to mistaken identity—a third person thought). As further discussed in Sect. 3.1.1,* de se* readings thus require some additional mechanism (e.g., descriptive readings of pronouns in Schlenker, 2004, challenged by Sharvit, 2008).

Even more problematically, third person pronouns in FID referring to the protagonist not only can, but must, in my judgment, be read *de se* (cf. Charnavel, 2019a, pp. 254–255). For (42) (with intended coreference between *his* and *John*) to be interpreted as FID, John must have recognized himself and been ready to say “my pants are on fire” (see (43)a). (42) can only render a third person thought if the FID passage does not convey that the narrator identifies the intended referent as the protagonist (see (43)b).

**Table:** 

(43)	a.	John_i_ was looking at himself in the mirror without recognizing himself. #*Oh his*_*i*_* pants were on fire!* John_i_ thought
	b.	John_i_ was looking at the mirror. *Oh his*_*k*_* pants were on fire*, John_i_ thought, *he*_*i*_* should help [this man]*_*k*_* as quickly as possible!* In fact, John was looking at no other than himself.

This judgment is confirmed by the observation that pronouns referring to the protagonist unbeknownst to the protagonist do not trigger Condition B effects when c-commanding pronouns knowingly referring to the protagonist as in (44) (inspired from Sharvit, 2008, p. 394 illustrating Condition C effects with pronouns referring to third party).

**Table:** 

(44)	When John_i_ arrived, Mary_k_ opened the door. *She*_*k*_* was a lab technician now. She*_*k*_* was holding the blood sample of [a man]*_*m*_* in her hand*. John_i_ got closer. *Could he*_*i*_* look?* (he asked) Inadvertently, he_i_ touched the blood. *Oh no!* (he exclaimed)* Did she*_*k*_* think he*_*m*_* could have infected him*_*i*_*? Of course not!* she answered, *the blood was his.*

This again supports ID-based analyses of FID. Certainly, a third person pronoun can, but need not be read *de se* in ID: as is well known, (45) can report a first person thought as well as a third person thought.^11^

**Table:** 

(45)	John_i_ thought that his_i_ pants were on fire.

But as is well known too, there exist some—so-called logophoric—elements specific to ID that must be read *de se*. As we will see in the next subsection, this is for instance the case of the possessive reflexive *his own*, whose use in (45) (instead of *his*) obligatorily gives rise to a *de se* reading. The fact that phenomena specific to ID are also attested in FID support an embedded analysis of FID.

Another similar fact in the tense domain corroborates such an approach. As observed by Sharvit (2008, p. 358), a language exhibits sequence of tense effects in FID if and only if it does in ID. For example, English and French exhibit sequence of tense effects both in ID and FID (see (46)), while Hebrew and Russian do not in either (see (47)).^12^

**Table:** 

(46)	a.	Two years ago John found out that Mary {was/#is} pregnant.				
	b.	Yes, she {#is/was} definitely pregnant(, thought Mary). (Sharvit, 2008, p. 356)				
(47)	a.	Yosef gila	Se	Miriam ohevet	et	Dan
		Yosef	find-out-PAST that	Miriam love-PRES	ACC	Dan
		‘Yosef found out that Miriam loved Dan.’				
	b.	Ken,	hi	le-lo safek ohevet	et	Dan(, xaSva Meri).
		Yes,	she definitely	love-PRES	ACC	Dan think-PAST Mary
		‘Yes, she definitely loved Dan(, Mary thought).’				(Sharvit, 2008, p. 357)

These facts directly derive from an ID-based analysis of FID (see Sharvit, 2008; cf. von Stechow, 2003), but must be stipulated under a DD-based analysis.

Furthermore, Sharvit (2008, p. 372) shows that tenses must be interpreted related to the protagonist in FID.^13^ The Hebrew past tense is unacceptable in (48) under a scenario where Dan wakes up from a coma in February and his wife was supposed to give birth on January 31, while he thinks it’s still January because the hospital staff forgot to turn the calendar page.

**Table:** 

(48)	*Mira*	*(#hayta) amura*	*laledet*	*be-yanuar*(, xaSav Dan)
	Mira	PRES/#PAST-be supposed	give-birth in-January	think-PAST Dan
	‘*Mira {is/was} supposed to give birth on January 31*(, Dan thought).’			(Sharvit, 2008, p. 372)

In sum, FID patterns like ID regarding an array of facts about pronouns and tenses, thus supporting an analysis treating FID as embedded discourse. Furthermore, we observe in FID some phenomena that are specific to embedded, so-called logophoric, discourse, which supports our hypothesis that FID is a kind of logophoric context. The next section details the latter class of facts.

#### Hallmarks of embedded contexts: logophors

A strong argument for the analysis of FID as a logophoric context relies on the availability in FID of logophoric elements anchored to the FID protagonist. The specificity of these elements is to refer to the perspective center of their context if (s)he is different from the speaker (see, e.g., Charnavel, 2021 for a review). The notion originated for some pronouns in West African languages that are reported to mainly occur in ID, in which they must refer to the attitude holder of that discourse (see Clements, 1975, i.a.). Logophoric pronouns are not attested in the languages under investigation here (see predictions for other languages in Sect. 2.4), but some elements of these languages exhibit logophoric properties. This is for example the case of exempt reflexives. As reviewed in, e.g., Charnavel (2019a), reflexives like *herself* or *her own* must descriptively be locally bound (in the relevant sense defined by Condition A, see, e.g., Charnavel & Sportiche, 2016) unless they are logophorically interpreted, as shown by the contrast in (49).

**Table:** 

(49)	a.	*[The iPhone broken into by the FBI]_i_ showed that private hackers had the ability to crack encrypted devices like itself_i_.
	b.	[The reality show chef]_i_ guessed that the next challenge would involve other chefs like himself_i_.
		(Charnavel, 2019a, p. 46)

Descriptively, neither the inanimate *itself* in (49)a, nor the animate *himself* in (49)b obeys Condition A, as their antecedent sits outside their clause. But only *himself* is acceptable (i.e., can be exempt from Condition A) because it can refer to the perspective center of its context, namely the attitude holder *the reality show chef*; *itself*, however, cannot refer to a perspective center, as inanimates cannot hold perspective (see Charnavel & Sportiche, 2016).

Crucially, exempt reflexives are attested in FID as illustrated in (50)–(51) (see also Fludernik 1993, pp. 125–126).

**Table:** 

(50)	a.	*That was one of the bonds between Sally and himself*. [Woolf, *Mrs Dalloway*]
	b.	*He still belonged to herself*, she believed. [Lawrence, *Sons and Lovers*]
	c	*And this was a bit destressing to people who did not share it; to Mr Carmichael perhaps, to herself certainly*. [Woolf, *To the Lighthouse*] (Banfield, 1982, p. 91)
(51)		Eve_i_ était très inquiète. *Comment allait-elle faire ? Ses*_*i*_* propres enfants et ceux du voisin refusaient de l’écouter depuis hier*.
		‘Eve_i_ was very worried. *How would she manage? Her*_*i*_* own children and the neighbor’s had been refusing to listen to her since yesterday*.’
		(Charnavel, 2020, p. 686)

These facts directly follow from an ID-based analysis treating FID as a logophoric context. The distribution and interpretation of exempt reflexives can generally be explained by the hypothesis that they are locally bound by a logophoric operator (see, e.g., Charnavel, 2019a, 2020, 2021). Similarly, the log-FID hypothesis directly accounts for (50)-(51), as will be detailed in Sect. 2.2. These facts, however, challenge DD-based analyses. Certainly, first person exempt reflexives can appear in DD such as (52), but it is far from straightforward to explain how they can appear at the third person in FID under both quotation or bicontextual accounts (see details in Sect. 3).

**Table:** 

(52)	This is one of the bonds between Sally and myself.

Some constraints on non-exempt uses of reflexives discussed in Sharvit (2010) and Sportiche (2022) corroborate the logophoric status of FID. Heim (1994) observes that the reflexive *himself* locally bound by PRO is acceptable in (53), even if *himself* is read *de re* and PRO is read *de se*.

**Table:** 

(53)	Oedipus wants PRO to find himself.

Let’s consider Oedipus’ myth according to which Oedipus is not aware of the fact that he killed his father himself, and he wants to find his father’s killer. In this context, PRO refers to who Oedipus takes himself to be, while *himself* refers to who the speaker (vs. Oedipus) takes Oedipus to be. To explain why Condition A is nevertheless satisfied, Sportiche (2022) proposes relativizing the binding conditions to some relevant attitude holder (see other arguments in Sportiche, 2022): *himself* in (53) is acceptable because it is co-valued with *PRO* at least for one attitude holder,i.e., for the speaker; in other words, Condition A is satisfied *de re*.

Now interestingly, a reflexive bound *de re* is unacceptable in FID as observed by Sharvit (2010):

**Table:** 

(54)		Mary was listening to an old recording of hers on the radio, not recognizing her own voice. The radio suddenly stopped playing, and she was disappointed that couldn’t make it play again.
	a.	She thought: ‘‘Ah! To hear this woman sing again!’’
	b.	She wanted to hear herself sing again.
	c.	Her mind was filled with frustrating thoughts. *Ah! To hear herself sing again!
		(Sharvit, 2010, p. 85)

Here, *herself* is acceptable in ID in (54)b, but unacceptable in FID in (54)c. This fact is difficult to derive without stipulation under quotation or bicontextual accounts, which must allow pronouns to be anchored to the speaker. However, as shown by Sportiche (2022), this fact directly follows from a logophoric analysis of FID, where everything—including Condition A—must be *de dicto*.^14^

**Table:** 

(viii)	A month ago, when I woke up from a coma, I didn’t know who I was. I listened to an old recording of myself, not recognizing my own voice. Suddenly, the radio stopped playing, and I was frustrated that I couldn’t make it play again. My mind was filled with frustrating thoughts. *Ah! To hear myself sing again!*

Another type of expression supporting the logophoric status of FID is motion verbs like *come*. This verb requires that a perspective center (e.g., speaker, addressee, attitude holder, empathy locus) be located at or associated with the goal of motion (see Oshima, 2006; Barlew, 2017; Charnavel, 2018, 2019a, i.a.). As expected under the log-FID hypothesis, *come* can be anchored to the protagonist or her addressee in FID.

**Table:** 

(55)	Harriet had begun to be sensible of his talking to her much more than he had been used to do […] *When they had been all walking together, he had so often come and walked by her, and talked so very delightfully*! [Jane Austen, *Emma*] (Anderson, 2022)
(56)	Richard turned to Lady Bruton, with his hat in his hand, and said, “We shall see you at our party to-night?” whereupon Lady Bruton resumed the magnificence which letter-writing had shattered. *She might come; or she might not come*. [*Mrs. Dalloway*, Virginia Woolf] (Anderson, 2022)

These facts are compatible with both ID-based and DD-based analyses as indicated by the acceptability of *come* in both ID and DD counterparts.

**Table:** 

(57)	a.	Harriet was glad that he had come and walked by her.
	b.	Lady Bruton replied to Richard that she might come.
(58)	a.	He has come and walked by me.
	b.	I might come.

However, *come* cannot be anchored to the narrator at the utterance time.

**Table:** 

(59)	Twelve years ago I had sat in my dreary London apartment, dreaming of Southern Italy, and now I was here. *Oh how lovely it would be there! To feel the sun and taste the food! If only I could one day earn enough to {go/#come} there!* So I had sighed then. (Anderson, 2022)

This fact challenges DD-based analyses (cf. Anderson, 2022): it is not fully clear how mechanisms designed to shift tense and pronouns to the narrator under these accounts (unquotation, presupposition anchoring to the narrator’s context) cannot also shift *come* here. However, this follows from the log-FID hypothesis because perspectival elements must be anchored to the logophoric center in logophoric contexts.^15^

Conversely, we observe that antilogophoric elements are banned from FID, which further supports the log-FID hypothesis against DD-based accounts. Epithets like *the idiot* have been argued to be antilogophoric in the sense that they cannot be used to refer to the logophoric center of their context (see Ruwet, 1990; Dubinsky & Hamilton, 1998; Patel-Grosz, 2012; Yashima, 2015, i.a.). More specifically, Charnavel (2019a, p. 145) shows that epithets are anti-attitudinal in the following sense: “regardless of who evaluates its content, an epithet occurring in an attitude clause cannot refer to the attitude holder of that clause unless it is read non *de se*.”

**Table:** 

(60)	a.	#John_i_ told us of a man who was trying to give [the idiot]_i_ directions.
	b.	John_i_ ran over a man who was trying to give [the idiot]_i_ directions.
	c.	#According to John_i_, [the idiot]_i_ is married to a genius
	d.	Speaking of John_i_, [the idiot]_i_ is married to a genius (Dubinsky & Hamilton, 1998, p. 688)
(61)		[*John hears on tape several people’s voices. He must determine whose voice could be used for some advertisement. He finds that a certain person’s voice sounds too aggressive for the task, without realizing that the person in question is John himself.]*
		John_i_ is convinced that [the idiot]_i_’s voice is too aggressive. (Schlenker, 1999, p. 78)

For example, *the idiot* cannot refer to John in (60)a,c, where it occurs in an attitude context anchored to John (if there is no mistaken identity about John, and even if John judges himself to be an idiot). However, *the idiot* can refer to John in (60)b,d, where it occurs in a context anchored to the speaker; note that this is the case in (60)b even if *the idiot* is c-commanded by John, which reveals that epithets are not subject to Condition C (but they are subject to Condition B, see, e.g., Dubinsky & Hamilton, 1998; Patel-Grosz, 2012). Furthermore, *the idiot* can refer to John in (61) where it is not read *de se*.

Crucially, epithets referring to the FID protagonist are unacceptable as shown in (62).

**Table:** 

(62)	Lucy_i_ was very worried. [The poor woman]_*i/k_*’s parents would not listen to her*.
	(Charnavel, 2019a, p. 147)

Even if Lucy considers herself to be a poor woman who should be empathized with, the second sentence in (62) cannot be interpreted as FID reflecting Lucy’s thoughts if *the poor woman* is intended to refer to Lucy. This fact directly derives from the log-FID hypothesis. Under DD-based hypotheses, this fact does not straightforwardly follow, as detailed in Sect. 3 (because of the availability of unquotation and because the epithet can correspond to the protagonist’s judgment).

Pronominal antilogophoric elements challenge DD-based accounts even more clearly. As observed by Ruwet (1990), the distribution of French prepositional clitics *en* and *y* is subject to the same constraint as epithets (see also *ce* in Coppieters, 1982 and *le* clustered with *lui* in Charnavel & Mateu, 2015): they cannot refer to the attitude holder of their contexts as exemplified in (63) (see review in Charnavel, 2019a, p. 148).

**Table:** 

(63)	a.	Emile_i_ pense que Sophie_m_ en_*i/k_ est amoureuse.
		‘Emile_i_ thinks that Sophie_m_ is in love with him_*i/k_.’
	b.	Emile_i_ pense que Sophie_m_ est amoureuse de lui_i/k_.
		‘Emile_i_ thinks that Sophie_m_ is in love with him_*i/k_.’
	c.	Emile_i_ mérite que Sophie_m_ en_i/k_ tombe amoureuse.
		‘Emile_i_ deserves Sophie_m_ falling in love with him_i/k_.’ (Ruwet, 1990, pp. 51, 53, 55)

The prepositional clitic *en* (‘of him’) cannot refer to Emile in (63)a, where Emile is the attitude holder of the clause containing *en*, while the strong pronoun *lui* is available in (63)b; however, *en* is acceptable in a non-attitude context, as in (63)c.

Strikingly, *en* is unavailable in FID to refer to the protagonist, as illustrated in (64).^16^

**Table:** 

(64)	a.	Emile_i_ exultait. *Oui, c’était sûr maintenant, Sophie*_*m*_ en_*i/k_ *était amoureuse!*
	b.	Emile_i_ exultait. *Oui, c’était sûr maintenant, Sophie*_*m*_* était amoureuse de lui*_*i/k*_*!*
		‘Emile_i_ was exulting. *Yes, it was now certain, Sophie*_*m*_* was in love with him*_*i/k*_*!*’

Hypotheses treating FID as a kind of attitude context (such as the log-FID hypothesis) can directly derive this fact. DD-based hypotheses, however, cannot, given that as we saw in Sect. 1.2.1, they must allow for pronouns to be anchored to the narrator (through unquotation or presupposition anchoring to the narrator).

Furthermore, it is not only in the person domain, but also in the tense domain that FID exhibits logophoric properties. This is especially the case of the German *Konjunktiv I* or reportive subjunctive (see Schlenker, 2003; von Stechow, 2003; Fabricius-Hansen & Sæbø 2004; Eckardt, 2014, i.a.). As described in Fabricius-Hansen & Sæbø (2004, p. 228), the German reportive subjunctive clause “is (in the same sentence or in the preceding context) the object of a verb of saying (claiming, asking, commanding) or it is understood as if it were”. The last part of this sentence is intended to capture cases of FID like (66). Otherwise, the German reportive subjunctive occurs in ID like (65).

**Table:** 

(65)	Er behauptete, dass jemand das Auto angefahren habe, . . .
	‘He claimed that somebody had driven into the car, . . .’ (Fabricius-Hansen & Sæbø 2004, p. 213)
(66)	Wedells Verteidiger Mario Ortiz gab sich optimistisch. *Der angebliche Beweis gegen seinen Mandanten reiche zu seiner Verurteilung bestimmt nicht aus*.
	‘Wedell’s counsel Mario Ortiz gave a show of optimism: According to him, the alleged proof against his client was definitely insufficient for a sentence. (Fabricius-Hansen & Sæbø 2004, p, 226)

The German reportive subjunctive thus appears in reported speech (in the complement of speech verbs) or in FID representing speech. Therefore, the occurrence of German *Konjunktiv* in FID directly follows under the log-FID hypothesis. In fact, Schlenker (2003) (cf. von Stechow, 2003) proposes that it is a logophoric mood. However, the appearance of German *Konjunktiv I* in FID is unexpected and requires some stipulation under DD-based analyses (see Eckardt, 2014; Maier, 2015) as detailed in Sect. 3.

In sum, FID is characterized by the fact that logophoric elements anchored to the protagonist are licensed in it, while antilogophoric elements are banned from it. These facts strongly support the log-FID hypothesis against DD-based analyses.

#### Recursive embedding

Another strong, new argument for analyzing FID as a kind of logophoric context is the possibility of multiple FID embedding. Recursive FID embedding has been sporadically noticed (implicitly by Banfield, 1982, pp. 130-131, explicitly by Doron, 1991, p. 63 or Eckardt, 2014, pp. 57-59, 250-251; see also Philippe, 2005, i.a.), but never taken into account in previous analyses, as acknowledged by Doron (1991) and Eckardt (2014).

A very clear example of double FID embedding is provided in (67).

**Table:** 

(67)	*Letting herself be helped by him*, Mrs. Ramsay had thought (Lili supposed) *the time has come now, she would say it now. Yes, she would marry him. And she stepped slowly, quietly on shore. Probably she said one word only, letting her hand rest still in his. I will marry you, she might have said, with her hand in his; but no more. Time after time the same thrill had passed between them—obviously it had*, Lily thought, smoothing a way for her ants. [Woolf, *To the Lighthouse*]
	(Doron, 1991, p. 63, noted as p.c. by Anita Mittwoch; Eckardt, 2014, pp. 250–251)

Here, the parentheticals *Mrs. Ramsay had thought (Lili supposed)* explicitly indicate the embedding of Mrs. Ramsay’s thoughts (shown in underlined italics) in Lili’s (shown in non-underlined italics). For instance, the indexical *now* (in *she would say it now*^17^) is anchored to Mrs. Ramsay’s temporality, while the adverb *obviously* is relativized to Lili’s judgment.

Some examples provided by Banfield (1982) to show the possible presence of a listening consciousness (which she compares with echo questions), such as (68), also seem to correspond to recursive FID:

**Table:** 

(68)	“Let us all go to the circus.” *No. He could not say it right. He could not feel it right*. *But why not?* she wondered. […] *Had they not been taken*, she asked, *to circuses when they were children? Never*, he answered. […] *He could never “return hospitality”* (*those were his parched stiff words*) *at college*. […] *He worked hard—seven hours a day; his subject was now the influence of something upon somebody*—they were walking on and Mrs. Ramsay did not quite catch the meaning, only the words, here and there . . . dissertation . . . fellowship . . . readership . . . lectureship.
	[Woolf, *To the Lighthouse*] (Banfield, 1982, pp. 130–131)

(68) reports a dialog between Mrs. Ramsay and Charles Tansley from Mrs. Ramsay’s perspective. Not only are Mrs. Ramsay’s thoughts (e.g., *No. He could not say it right. He could not feel it right*) and speech (e.g., *But why not?*) reported in FID, but Charles Tansley’s represented speech is also embedded within Mrs. Ramsay’s FID, as clearly indicated by the following: Mrs. Ramsay’s FID includes comments on Tansley’s choice of words (*those were his parched stiff words*), and Tansley’s last sentence (*his subject was the influence of something upon somebody*) is clearly represented through Mrs. Ramsay’s consciousness that does not identify the content of the dissertation’s subject, as then explicitly stated by the rest of the narration (*Mrs. Ramsay did not quite catch the meaning, only the words*). In other words, just like (67), (68) can be transposed into ID using two attitude embeddings, the only difference consisting in the nature of the higher attitude (perception vs. thought).

**Table:** 

(69)	a.	Lily supposed that Mrs. Ramsay thought that she would marry him.
	b.	Mrs. Ramsay heard that Charles Tansley said that his subject was the influence of something upon somebody.

Moreover, Doron (1991) reports a passage (without analyzing it) that seems to include recursive FID anchored to the same protagonist (at different times).

**Table:** 

(70)	*It was in all this she had found her occasion. She would launch his boat for him; she would be his providence; it would be a good thing to love him. And she had loved him* [...].
	[Henry James, *The Portrait of a Lady*] (Doron, 1991, p. 63, noted as p.c. by Moshe Ron)

(70) is an excerpt from Isabel’s meditations, in which she reflects on how she was seduced by Osmond. The contrast between the past perfect (*had found, had loved*) and the future in the past (*would*) indicates that Isabel’s past thoughts are embedded within her present thoughts, as can be transposed in the following ID:

**Table:** 

(71)	Isabel thought that she had thought that it would be a good thing to love him.

These few examples thus reveal that FID can be recursively embedded under various configurations. As acknowledged by Eckardt (2014, pp. 58–59, 250–251), this fact challenges DD-based analyses, especially bicontextual analyses (mixed quotation accounts are challenged to a lesser extent; see details in Sect. 3). However, it can be explained by the log-FID hypothesis, which is compatible with the stacking of logophoric operators (provided that semantico-pragmatic conditions are satisfied, as discussed in Sect. 2). This fact thus strongly favors ID-based against DD-based analyses.

Furthermore, this fact suggests a partial explanation for the debated distribution of first person pronouns in FID (see Sect. 1.2.1). Recall that while Banfield (1982) claims that first person pronouns can only appear in FID if referring to the protagonist or her addressee, Schlenker (2004) contends that their distribution is regulated by the pragmatics of narration: they are acceptable in FID as long as the narrator presents herself as taking part to the action she is reporting and cannot thus be an omniscient narrator; there must therefore be a plausible reason for her to have information about the represented speech or thoughts. But the examples (21)-(22) Schlenker (2004) provides (repeated in (72)-(73)) do not seem to fully satisfy these conditions.

**Table:** 

(72)	a.	*Oh how extraordinarily nice I was*, she told my father, without realizing that I was listening to their conversation.
	b.	[Reporting on thoughts I read in my mother’s diary]. *Oh how extraordinarily nice I was*, she thought.
(73)		I had become adept at reading my teachers’ thoughts. My Greek teacher didn’t like me so much after all. *Really, I was a little devil, not entirely without talent, but impossible to deal with—something had to be done about me, or else tomorrow I would become completely unbearable*.

While the narrator is indeed presented as having direct evidence about the represented speech in (72)a (she is present during the reported conversation), she is only presented as having indirect and delayed evidence about it in (72)b (she reads about it after the narrated action) and the reason for her to have information about the represented thought in (73) does not appear to be plausible (she is supposed to have magical powers that allow her to read other people’s minds, while the narration is otherwise realistic and the narrator is not supposed to be omniscient). Interestingly, these problems disappear if we hypothesize that (72)-(73) are instances of recursive embedding as made explicit by the following ID counterparts:

**Table:** 

(74)	a.	I read that she thought that that I was extraordinarily nice.
	b.	I guessed that he thought that I was a little devil.

In fact, (73) is compatible with a motion verb anchored to the narrator, indicating that the narrator can indeed be construed as a logophoric center here:^18^

**Table:** 

(75)	I had become adept at reading my teachers’ thoughts. My Greek teacher didn’t like me so much after all. *Really, I was a little devil. Tomorrow, he would come to see me and talk to me*.

Note that this possibility further raises the question whether Banfield’s (1982) original intuition was not right after all, according to which first person pronouns are licensed in FID only if the narrator is presented as a FID protagonist or her addressee (cf. Doron, 1991; Cumming & Sharvit 2016). Examples (72)a and (20), where the narrator is present in the narrated action, could also lend themselves to this analysis:

**Table:** 

(76)	a.	I heard that she told my father that I was extraordinarily nice.
	b.	I heard that Ralph asked me if I really knew the road.

But just as the discussion about the second person suggested in Sect. 1.2.1, this constraint seems to only hold if FID occurs in a fictive narration (where the narrator’s context is not precisely identified, see further discussion in Sect. 2.3). If the FID occurs in a standard discourse involving a speaker and an addressee, it seems that the speaker can be referred to by a first person pronoun in FID without being construed as a logophoric center.

**Table:** 

(77)	(You know what?) When my mother was pregnant with me, she often dreamt about when I would arrive. *How lovely it would be to look at my little eyes!*

The first person possessive *my* is here acceptable in the FID fragment anchored to the speaker’s mother. But at the time of the narrated action, the speaker (still a fetus) cannot formulate any thought and cannot thus be a perspective center. Of course, the narrator must have been informed at some point by her mother about these thoughts to be able to report them (since (77) is intended not to be from fictive narration) so that we could in principle reconstruct a double attitude embedding:

**Table:** 

(78)	I heard from my mother that she had thought that it would be lovely to look at my little eyes.

But it seems that the discourse in (77) can be understood as directly focusing on the narrator’s mother thoughts, making the source of the information irrelevant, just as in the ID counterpart in (79).

**Table:** 

(79)	My mother thought that it would be lovely to look at my little eyes.

Similarly, a second person pronoun can appear in FID if (and only if) it refers to the actual addressee of the speaker. Crucially, it need not be construed as protagonist. The only requirement is that the speaker have the relevant information to present the actual addressee as a participant in the narrated action (e.g., by taking part in the action herself as protagonist or addressee as in (80)-(81) or by having some evidence about it).

**Table:** 

(80)	a.	[cf. (28)] We were all worried about you and talked about it in your absence. *What would you become after this?*’
	b.	We wondered what you would become.
(81)	a.	[= (2)] X has called. *Can we drive him, you or me, to Roissy at 5am tomorrow?* He is dreaming!
	b.	X asked me if you or I can drive him to Roissy.

In sum, the possibility of recursive FID embedding not only argues against DD-based analyses, but also clarifies some aspects of the distribution of first person pronouns in FID. Due to recursive embedding, first person pronouns in fictive narration can be assumed to only refer to the FID protagonist or her addressee. It is only in communicative (well-defined) contexts that first and second person pronouns need not refer to the FID protagonist or her addressee, as long as the speaker has appropriate (direct or indirect) evidence for presenting herself or her addressee in the represented speech or thought.

#### Style

The last argument against DD-based analyses relies on stylistic considerations. When discussing example (13), we have observed that FID can reflect the manner of speaking of the protagonist just as is the case in DD. But crucially, FID need not be faithful to the speech or thought that it represents in ways that challenge DD-based analyses (*pace* Schlenker, 2004, p. 285; Maier, 2015, p. 357).

As noticed in, e.g., Banfield (1982, pp. 114–116), Fludernik (1993, Ch. 8) or Abrusán (2023, pp. 17–18), FID need not be a verbatim report of an actual speech or thought act. Neither pronunciation (phonetic properties), nor style (syntactic and vocabulary choices) need be identical to the thought or speech that is intended to be represented.^19^ As argued by Fludernik (1993), this is especially evident when FID does not reflect one speech or thought act, but condenses several ones, whether by the same protagonist at recurrent times, or by several protagonists through plural FID (see also Abrusán, 2023) as illustrated in (82).

**Table:** 

(82)	If I told my schoolmates that Lena Lingard’s grandfather was a clergyman, and much respected in Norway, they looked at me blankly. *What did it matter? All foreigners were ignorant people who couldn't speak English*. (Fludernik, 1993, p. 399; Abrusán, 2023, p. 18)

This typicality and schematic nature of FID does not, however, straightforwardly argue against DD-based analyses. As also argued by Fludernik (1993, Ch. 8), DD need not be an exact report of some original discourse. In fact, the transposition of (82) into DD does not require any reformulation (except for tense, of course, as discussed above).

**Table:** 

(83)	If I told my schoolmates that Lena Lingard’s grandfather was a clergyman, and much respected in Norway, they looked at me blankly and said: “What does it matter? All foreigners are ignorant people who can't speak English.”

But crucially, FID can also involve some reformulation that is incompatible with DD. First, the choice of words can be explicitly indicated as different from the original discourse as in (84).

**Table:** 

(84)	Quelque temps après [F. Mitterrand] me demanda de l’aller voir. […] Il me dit qu’il prêtait attention à ce que j’écrivais, à mon ton, à mon style… *Serais-je intéressé d’être mêlé à son environnement* (il employa un autre mot que j’ai oublié, car celui-ci n’existait pas à cette époque) *politique ?* Non, je n’avais de goût que pour l’écriture. [J. Cau, *Croquis de mémoire*, 1986] (Authier-Revuz, 2020, p. 315)
	‘After some time, [Mitterrand] asked me to come see him. […] He told me that he paid attention to what I wrote, to my tone, my style. *Would I be interested in being involved in his political environment* (he used another word I forgot, as this one did not exist at that time).’

Here, the word *environnement* is used in FID although it is explicitly mentioned as being absent from Mitterrand’s actual discourse. This is infelicitous in DD:

**Table:** 

(85)	#F. Mitterrand me demanda (en utilisant un autre terme): « Seriez-vous intéressé d’être mêlé à mon environnement politique ? »
	‘#F. Mitterrand asked me (using another word): “Would you be interested in being involved in my political environment?”

Second, the style in FID can be clearly distinct from the expected style of the protagonist, while DD is expected to reproduce it. This is for instance the case of example (19) relating Proust’s thoughts when he was a child: the style does not reflect a child’s manner of speaking (which would be used in DD), but is rather the narrator’s. Joyce’s following example makes the point even more clearly:

**Table:** 

(86)	At what o’clock did you dine? he [Bloom] questioned of the slim form and tired though unwrinkled face.
	—Some time yesterday, Stephen said.
	—Yesterday! exclaimed Bloom till he remembered it was already tomorrow, Friday. Ah, you mean it’s after twelve!
	—The day before yesterday, Stephen said, improving on himself. […]
	*Anyhow, upon weighing the pros and cons, getting on for one, as it was, it was high time to be retiring for the night. The crux was it was a bit risky to bring him [Stephen] home as eventualities might possibly ensue (somebody having a temper of her own sometimes) and spoil the hash altogether as on the night he misguidedly brought home a dog (breed unknown) with a lame paw, not that the cases were either identical or the reverse though he had hurt his hand too) to Ontario Terrace as he very distinctly remembered, having been there, so to speak*. [Joyce,* Ulysses*](Fludernik, 1993, p. 392)

As argued by Fludernik (1993, p. 392), the comparison between Bloom’s DD and FID makes obvious the stylistic contrast between the two: while DD seems to be intended to truthfully represent Bloom’s manner of speaking, FID seems to be written in the narrator’s style.^20^

In sum, the style used in FID is not intended to faithfully report the manner of speaking as in DD (even under the assumption that a faithful report is not a verbatim report). This fact challenges DD-based analyses.

### Back to similarities to DD

We have thus observed in FID several properties that argue for an ID-based, logophoric analysis, against DD-based analyses; this point will be made more precise in Sects. 2 and 3 when examining specific implementations of logophoric and DD-based analyses. In the rest of this section, we reexamine the properties described in Sect. 1.1 that are supposed to argue for DD-based analyses against ID-based analyses and show that they do not, after all, challenge a logophoric, ID-based analysis.

#### Root clause syntax

The property that is usually first mentioned against ID-based analyses is the fact that FID exhibits root clause syntax: all characteristics of syntactic embedding shown in ID are precluded in FID, as seen in Sect. 1.1.1. But syntactic properties of (attitude) embedding should be distinguished from semantic ones. For example, (87) (cf. (1)) expresses Emma’s attitude without showing the syntax of embedded clauses.

**Table:** 

(87)	Selon Emma, Lheureux voulait sans doute l’effrayer encore.
	‘According to Emma, Lheureux probably wanted to scare her again.’

This is due to the fact that *selon Emma* ‘according to Emma’ semantically introduces another attitude than the speaker’s without syntactically embedding a clause.^21^ Similarly, we can assume that FID is introduced by an implicit operator that behaves like an attitude verb semantically, but not syntactically. This is the case of Sharvit’s (2008) FID operator (cf. Giorgi, 2010).^22^ This will also be the case of our log-FID operator.

#### Protagonist’s privileged perspective

As seen in Sect. 1.1.2, DD-based analyses are also supported by the fact that FID mainly presents the protagonist’s perspective: in particular, *de re* non *de dicto* readings are prohibited in FID and indexicals are anchored to the protagonist. The goal of this section is to show that these points do not, after all, argue against a logophoric ID analysis.

First, it is important to note that ID does license *de dicto* readings. This is uncontroversially the case of referential expressions like *his mother* (see (8)a), but also the case of evaluative expressions like epithets (see, e.g., Patel-Grosz, 2012), expressives (see, e.g., Schlenker, 2007), speaker-oriented adverbs (see, e.g., Giorgi, 2010, pp. 72–75) or discourse particles (see, e.g., Zimmermann, 2011), as well as style or dialect, as shown in (88)–(92).

**Table:** 

(88)	I am not prejudiced against Caucasians. But John, who is, thinks/claims that you are the worst honky he knows. (Schlenker, 2003)
(89)	My father screamed that he would never allow me to marry that bastard Webster.
	(Kratzer, 1999, cited by Schlenker, 2007)
(90)	Mario disse a tutti che francamente era stanco di ascoltare sciocchezze. [Italian]
	‘Mario told everybody that frankly he was tired of hearing silly things.’ (Giorgi, 2010, p. 73)
(91)	Tom glaubt, dass wohl die Birne durchgebrannt ist. [German]
	‘Tom believes that wohl the bulb has burned out.’ (Eckardt, 2014, p. 129)
(92)	[…] to protest, like the Japanese in the anecdote, that he was altogether flummoxed and perplexed by position of Honorable Bird. [Huxley, *After Many a Summer*] (Fludernik, 1993, p. 255)

The argument against ID-based analyses thus lies on the obligatoriness of *de dicto* readings, since ID usually allows for, but does not require *de dicto* readings.

But crucially, the obligatoriness of *de dicto* readings is not in principle incompatible with attitude embedding. Sharvit’s FID operator achieves obligatory *de dicto* readings by positing quantification over context-assignment pairs. Independently of FID, Charnavel’s (2019a) logophoric operator is also designed to force *de dicto* readings under its scope; this is motivated by the fact that logophoric reflexives cannot co-occur with non *de dicto* expressions in the same local domain as illustrated in (93).

**Table:** 

(93)	*[My grandfather Joseph mistakes fuzzy photos of me (taken from behind) for old portraits of himself and finds them beautiful while I think they are horrible.]* (Charnavel, 2019a, p. 160)
	Joseph_i_ espère que les affreuses photos de lui_i_(*-même) sont préservées de la lumière.
	‘Joseph_i_ hopes that the horrible photos of him_i_(*self) are protected from light.’

The obligatoriness of *de dicto* readings is thus not only compatible with ID-based analyses, but it directly follows from a logophoric analysis (see further details in Sect. 2.2).

The second argument based on adverbial indexicals, which is one of the main arguments motivating DD-based analyses, is also to be qualified. As mentioned in Sect. 1.1.2, it is usually stated that unlike in ID, adverbial indexicals are not anchored to the speaker, but to the protagonist in FID, just like in DD.^23^ But several aspects of this statement need to be refined. First, it is in fact not the case that location and time indexicals are necessarily anchored to the speaker outside FID and DD, even in languages that do not exhibit indexical shift. One reason for this is that some adverbial indexicals, such as English *now* and *here* (see Kamp & Reyle, 1993; Recanati, 2004; Hunter, 2012; Altshuler, 2016; Lee, 2017; Anand & Toosarvandani, 2019, i.a.), French *maintenant* and *aujourd’hui* (Gollut & Zufferey, 2021), or German *jetzt* and *hier* (Eckardt, 2014, pp. 44–45), have been observed not to be anchored to the utterance time even outside reported discourse contexts:

**Table:** 

(94)	In the summer of 1829, Aloysia Lange, née Weber, visited Mary Novello in her hotel room in Vienna... Aloysia, the once celebrated singer, now an old lady of sixty-seven... gave Mary the impression of a broken woman lamenting her fate. (Recanati, 2004, p. 18, adapted from Predelli, 1998b)
(95)	Napoleon wurde 1815 auf die Insel Sankt Helena verbannt. Jetzt war er nicht mehr so populär.
	‘Napoleon was banned to St. Helena in 1815. Now, he was not so popular anymore.’
	(Eckardt, 2014, pp. 44-45)
(96)	Notre héros eut la gaucherie de s’arrêter auprès de cette petite chaise de paille, qui jadis avait été le témoin de triomphes si brillants. Aujourd’hui, personne ne lui adressa la parole.
	‘Our hero had the awkwardness to stop beside this little straw chair, which had once witnessed such brilliant triumphs. Today, no one spoke to him.’
	[Stendhal, *le Rouge et le Noir*] (Gollut & Zufferey, 2021, p. 74)

Another reason is that even adverbial indexicals that do not exhibit this apparently non-indexical behavior can in fact be evaluated with respect to a reported context in ID as observed by, e.g., Plank (1986), Fludernik (1993) or Anderson (2019).

**Table:** 

(97)	Last week, Jane said that she would order the cake tomorrow, but she didn’t. (Anderson, 2019, p. 2)
(98)	Nevertheless he [Tom] submitted to be kissed willingly enough though Maggie hung on his neck in rather a strangling fashion, while his blue-grey eyes wandered towards the croft and the lambs and the river, where he promised himself that he would begin to fish the first thing tomorrow morning.
	[G. Eliot, *Mill on the Floss*] (Fludernik, 1993, p. 225)

Anderson (2019) experimentally shows that many American English speakers find (97) coherent, thus relativizing *tomorrow* to the reported speaker; (98) is a natural occurrence of this pattern.

Second, it is crucial to note that location and time adverbials need not be indexical in FID: they can also be anaphoric—a fact that is overlooked in the formal linguistic literature.^24^

**Table:** 

(99)	*Und wie er auch gebeten hatte, es nicht anzusagen, so hatte sie es doch an dem Tage noch ihrem Guten redlich gemeldet, weil er’s wissen muβte*.
	‘*And however much he had begged her not to, she had on that day honourably confessed to her good, upright bridegroom, who had to know*.’
	[Mann, *Lotte in Weimar*] (Fludernik, 1993, p. 225, quoted Weinrich, 1985, p. 242)
(100)	[…] enfin il fit valoir des raisons personnelles, *le mort était son beau-père, le beau-père du maire de Rognes. Voyons, ce serait pour le lendemain dix heures*.
	‘[…] finally he put forward personal reasons, *the dead man was his father-in-law, the father-in-law of the mayor of Rognes. Come on! it would be at ten o'clock the next day*’.
	[Zola, *La Terre*] (Authier-Revuz, 2020, p. 135; see also Gollut & Zufferey, 2021)

Such occurrences, which are unacceptable in DD, challenge DD-based analyses.^25^

Third, and most importantly, the behavior of adverbial indexicals is crucially different in FID occurring in communication (vs. narration), i.e., in the presence of well-defined speaker and addressee. According to Eckardt (2014, p. 217), their interpretation is ambiguous: *heute* in (101) can be anchored to Tom or the speaker.

**Table:** 

(101)	Tom hat letzte Woche angerufen. *Er komme heute*.
	‘Tom called last week. *He would come today*.’

According to Gollut and Zufferey (2021, p. 86), they must be anchored to the actual speaker: in (102), where the actual speaker is explicitly temporally located (see *il y a trois jours* ‘three days ago’), the protagonist’s temporality cannot be expressed by an indexical (*hier* ‘yesterday’), but only by an anaphoric adverbial (*la veille* ‘the day before’).

**Table:** 

(102)	X m’a téléphoné il y a trois jours pour m’expliquer les raisons de son absence: *il avait {#hier/la veille} contracté un gros rhume*. (Gollut & Zufferey, 2021, p. 86)
	‘X called me three days ago to explain the reasons for his absence: *{#yesterday/the day before} he had contracted a bad cold*.’

It is thus only when the speaker’s temporality coincides with the protagonist’s, as in (103), that adverbial indexicals can be used and refer to the protagonist’s circumstances in FID occurring in communication.

**Table:** 

(103)	Le cœur d’Edmond bat. […] *Allons, il faut sonner*. *Le silence et l’odeur des roses. La bonne*. « Mademoiselle a téléphoné. Elle s’excuse auprès de monsieur. *Si monsieur veut l’appeler demain matin à l’appareil… Pas trop tôt*… » [Aragon, *Les beaux quartiers*]
	‘Edmond’s heart beats. […] Come on, you have to ring the bell. The silence and the smell of roses. The maid. “*Mademoiselle* called. She apologizes to monsieur. *If monsieur wants to call him tomorrow morning on the telephone… Not too soon*…”’ (Gollut & Zufferey, 2021, p. 86)

In (103), *demain* ‘tomorrow’ occurs in FID anchored to Mademoiselle, whose temporality corresponds to that of the maid, which corresponds to the speaker here (FID is embedded within the maid’s DD).

Giorgi (2010, pp. 193–194) even strengthens this claim: according to her, adverbial indexicals must be anchored to the narrator’s (vs. the protagonist’s) temporality as long as the narrator is explicitly referred to in FID as shown by the contradictoriness of (104) (inspired from (72)).

**Table:** 

(104)	#*Oh how extraordinarily nice I was yesterday night*, she told my father last morning, without realizing that I was listening to the conversation.
	(Giorgi, 2010, p. 193)

In sum, the behavior of adverbial indexicals, which is usually treated as the main argument for DD-based analyses, is in fact far from straightforwardly supporting DD-based against ID-based analyses. Under DD-based analyses, the shiftability of adverbial indexicals in some IDs, the availability of anaphoric adverbials in FID and the interpretation of adverbial indexicals in FID occurring in communicative discourse, remain problematic. We will discuss in Sect. 2.3 how the log-FID analysis can fare better in this respect.

To recapitulate, we have observed in this section that a fine-grained description of FID properties as compared to DD and ID properties rehabilitates the class of ID-based analyses; on the contrary, the class of DD-based analyses, which have been favored in recent years, comes out undermined. In the next section (Sect. 2), I propose a specific implementation of ID-based analyses, i.e., the log-FID analysis, and show how it can derive the properties of FID.

## Proposal: the log-FID analysis

In the previous section, we have seen that several facts challenge DD-based analyses of FID, such as the (un)acceptability of (anti)logophoric elements anchored to the protagonist, the possibility of FID recursive embedding, *de se* and *de te* readings, number and gender pronominal interpretations, or the distribution of first and second person pronouns. For interested readers, Sect. 3 will detail the problems encountered by each main implementation of DD-based analyses, thus specifying the general points of Sect. 1.

The goal of this section is to instead propose an ID-based analysis of FID that meets empirical adequacy. As we will see, crucial aspects of the analysis are inspired by Sharvit’s (2008) proposal (itself partially inspired by Doron, 1991 and Schlenker, 1999,26): the main idea will be to unite Sharvit’s silent attitude operator with a logophoric operator. FID will thus be (correctly, as we will see) predicted to exhibit all the properties that logophoric contexts have been independently shown to exhibit (by Charnavel, 2019a, 2020, i.a.), i.e., obligatory *de dicto* readings, licensing of exempt anaphora and other logophoric elements, anti-licensing of anti-attitudinal and anti-logophoric elements, perspectival homogeneity and non-shifting of indexicals (as exemplified in Sects. 1.2.2, 1.3.2, 2.2, 2.3).

More specifically, like Sharvit (see also Cumming & Sharvit 2016), I will posit an implicit intensional operator scoping over FID passages and responsible for their *de se* and *de dicto* properties. We will see that this operator is further motivated by some of the new properties we described in Sect. 1, such as FID recursivity or the ban on anti-attitudinal elements. The main analytical innovation as compared to Sharvit will be to assimilate this operator to a logophoric operator, thereby classifying FID as a logophoric context. This log-FID operator will be designed to license logophoric properties (not only *de se* and *de dicto* readings like Sharvit’s FID operator, but also logophoric elements like exempt anaphors). On the basis of new empirical observations, I will further depart from most previous analyses by relaxing the dependency of adverbial indexicals on the protagonist’s context. A problematic ingredient of Sharvit’s analysis (which is claimed to make it hybrid and idiosyncratic) will thus be abandoned, namely dual context dependency and quantification over context. Instead, our FID operator will resemble the logophoric operators found outside FID contexts, and the peculiar use of some indexicals in FID (not only adverbial indexicals, but also first and second person pronouns) will be attributed to the specificity of fiction, which often hosts FID.

Section 2.1 motivates the presence of an attitudinal operator in FID. Section 2.2 shows that this operator must specifically be a logophoric operator. Section 2.3 provides an account for the behavior of indexicals. Section 2.4 addresses the remaining issues, including predictions to check and refinements to make in future research.

### An attitudinal operator

Under our log-FID account, the perspectival properties of FID are not captured by internal context dependency as in DD-based analyses, but by the presence of an implicit intensional operator as in Sharvit (2008) and Cumming and Sharvit (2016). The goal of this section is to motivate this move by showing how several (already observed as well as new) properties of FID can (only) be derived by such an operator. Note that as in Sharvit (2008), this operator is here hypothesized to share with overt attitude verbs (part of) their semantics (as will be further specified in Sect. 2.2), but not their syntax; this is why FID does not exhibit properties of syntactic embedding, as shown in Sects. 1.1.1 and 1.3.1.

The hypothesis of an implicit attitudinal operator scoping over FID passages correctly derives several properties of FID independently observed in attitude contexts. First, this hypothesis explains why the truth of the thought or speech in FID is attributed to the protagonist, and not to the narrator. In fact, even some DD-based analyses, namely mixed quotation analyses, need to posit such an operator (besides quotation devices) for this reason (see Maier, 2015, p. 368). Note that under bicontextual accounts, the correct truth attribution can be done pragmatically; specifically, Eckardt (2014) incorporates this aspect in the Stalnakerian pragmatic rules of context update she assumes. This aspect is thus not underivable in the absence of an attitude operator, but remains a (weak) argument for the operator hypothesis that straightforwardly derives it. Further note that the constraints on the protagonist’s identity depend on this accommodation of an implicit attitudinal operator (sometimes doubled with an overt parenthetical, see discussion in Sect. 2.4): the attitude operator takes a subject that must be identified with the relevant protagonist. This can be assumed to be done by usual semantico-pragmatic rules of coreference: the discourse preceding the FID passage makes salient the relevant protagonist, as illustrated in (105) representing (1).^27^

**Table:** 

(105)	pro_i_-logFID [Lheureux probably wanted to scare her_i_ again, she_i_ thought]

Second, the attitude operator hypothesis directly accounts for (anti)attitudinal properties of FID. On the one hand, as seen in Sect. 1.1.2, referential expressions are read *de dicto* in FID and similarly, evaluative expressions such as epithets, expressives, speaker-oriented adverbs or discourse particles are evaluated by the protagonist. Given that, as seen in Sect. 1.3.2, attitude contexts license *de dicto* readings and the relativization of evaluative expressions to their attitude holder, it follows that evaluative and referential expressions can be anchored to the protagonist in FID under our attitude operator hypothesis. In Sect. 2.2, we will further see how defining our attitude operator as a logophoric operator can derive why such anchoring is obligatory. On the other hand, we observed in Sect. 1.2.2 that anti-attitudinal elements anchored to the protagonist (such as epithets or some French pronouns) are banned from FID. This (so far overlooked) fact straightforwardly follows from our hypothesis since we independently observe that these elements are unacceptable in attitude contexts when anchored to the attitude holder associated with them (unless they are not read *de se*; but as we also saw in Sect. 1.2.1, non *de se* readings are precluded in FID).

**Table:** 

(106)	*pro_i_-logFID [Lheureux probably wanted to scare [the idiot]_i_ again, she_i_ thought]

This observation provides a strong argument for our operator hypothesis against DD-based analyses: while DD-based analyses straightforwardly derive obligatory attitudinal properties such as *de dicto* readings or perspectival interpretations aforementioned, they cannot derive the ban on anti-attitudinal expressions in FID, which are incorrectly predicted to be licensed by the mechanisms required for shifting tense and pronouns to the narrator (e.g., unquotation, presupposition anchoring to the narrator’s context).

Although we have to leave for further research (for lack of analytical tools) a detailed account of stylistic and dialectal aspects of FID here, note that the observations we made about manner of speaking in Sects. 1.1.2 and 1.2.4 seem to provide the same kind of argument for the operator hypothesis against DD-based accounts; only an attitudinal operator account can explain why not only the manner of speaking of the protagonist (given that, as noted by Sharvit (2008, p. 391), ID can incorporate quotative elements), but also, crucially, that of the narrator can be reflected in FID. Similarly, we observed in Sect. 1.2.1 that vocatives and (most) imperatives are banned from FID—a property that uniquely characterizes complements of attitude verbs. Even without providing a precise analysis of such facts (which are controversial and remain poorly understood),^28^ we can thus at least conclude that they are compatible with a treatment of FID as incorporating a silent attitudinal operator.

Third, the attitude operator hypothesis can uniquely derive *de se/de te* readings as well as sequence of tense phenomena. These are Sharvit’s (2008) main, crucial arguments for her FID operator against DD-based accounts (see also Cumming & Sharvit 2016), which I adopt here, along with her specific analysis of them. Recall from Sect. 1.2.1 that in FID, pronouns referring to the protagonist must be read *de se* (and pronouns referring to her addressee must be read *de te*), and a language exhibits sequence of tense effects in FID if and only if it does in ID. As acknowledged by most DD-based analyses, *de se* (and *de te*) readings and sequence of tense phenomena cannot be captured in the absence of an attitudinal operator. In fact, Maier (2015, fn. 39) does posit such an operator, and Schlenker admits that his e-type solution to *de se* readings is stipulative (while Eckardt, 2014 does not discuss these points). Instead, Sharvit (2008) and Cumming and Sharvit (2016) show how binding by an implicit attitudinal operator can derive these facts. To this end, they specifically adopt von Stechow’s (2003) mechanism of feature deletion under agreement. As Sharvit (2008, p. 359) explains: the idea is that a tense embedded under an agreeing tense may be interpreted as a zero-tense (i.e., a relative present; in ID and FID—the subject’s “now”), and that a personal pronoun embedded under an agreeing pronoun may be interpreted as a zero-person (i.e., a relative first person; in ID and FID—the subject’s “I”).

Concretely, past or *phi*-features can be deleted when the relevant tense or pronoun is bound by an attitude operator (including the FID operator) as schematized in (107)-(108) (cf. (46)-(47)). While (108) is available in both English and Hebrew, (107) is only available in English.

**Table:** 

(107)	a.	
	b.	
(108)	a.	
	b.	

More specifically, assuming like von Stechow that attitude operators quantify over individual-world-time triples (see further details in Sect. 2.2), binding of a person pronoun by the FID operator ensures that it refers to whoever the subject takes himself to be,^29^ and binding of a tense pronoun by the FID ensures that it refers to the time where the subject locates himself.

**Table:** 

(109)	John^3M^-logFID-Tns^PST^ λ<x^3M^, t^PST^, w> [x^3M^ t^PST^ a genius]

Further assuming like von Stechow that semantic binding requires agreement of features and that the features of bound variables must be deleted, it follows that the featural interpretation of *de se* pronouns and tenses is relativized to the narrator: their features are not interpreted in the attitude context (FID), but only on the arguments of the operator. Thus, a further virtue of this account as compared to alternative, DD-based ones, is that it can derive the complex judgments related to gender (and number) features in FID that we observed in Sect. 1.2.1: while the gender (and number) judgments for third party (excluding the protagonist’s addressee) are anchored to the protagonist, it is the narrator that is responsible for those for the protagonist (and her addressee). This latter point directly derives from the hypothesis that pronouns referring to the protagonist (or her addressee) are *de se* (or *de te*) pronouns subject to feature deletion (and the former point derives from the obligatoriness of *de dicto* readings that will be explained in Sect. 2.2).

Further note that the same holds for first and second person pronouns (cf. von Stechow, 2003; Sharvit, 2008, p. 390) as long as we assume a uniform semantics for all personal pronouns, i.e., a presuppositional semantics for person features as independently argued for (see Heim, 2008; Charnavel, 2019c, i.a.): as exemplified in (110) representing (27), the person features are not interpreted in FID when first or second person pronouns are *de se* (or *de te*) pronouns, i.e., when they refer to the protagonist (or her addressee); however, they are interpreted by the narrator (as the author or the addressee of the actual context, i.e., as the narrator or her addressee, as seen in Sect. 1.2.1). Note that the interpretation of first and second person pronouns interpretation when they are not *de se* (or *de te*) pronouns (i.e., outside fiction in communication contexts) will be discussed in Sect. 2.3.

**Table:** 

(110)	pro_i_^2nd^-logFID Remember, you_i_ were not good enough (you_i_ thought).
	LF: pro^2nd^-logFID λ<x^2nd^,..> Remember, x^2nd^ were not good enough

A fourth, strong argument for the attitude operator hypothesis relies on the observation in Sect. 1.2.3 that FID can be recursively embedded. This fact directly follows from an analysis involving attitude operators, which can in principle be stacked as schematized in (111) roughly representing (67). As acknowledged by Eckardt (2014, pp. 250–251) and further detailed in Sect. 3, thought or speech embedding is however underivable under bicontextual accounts.

**Table:** 

(111)	[pro_i_-logFID [pro_k_-logFID *Letting herself be helped by him*, Mrs. Ramsay_k_ had thought (Lili_i_ supposed) *the time has come now, she would say it now*.]]

Furthermore, recall from Sect. 1.2.3 that the embeddability hypothesis allows for a plausible account for the distribution of first and second person pronouns in FID in fiction, thus providing an indirect argument for positing attitude operators in FID. For instance, we can assume that (73) can include a first person pronoun because the narrator can be construed as the subject of a silent attitudinal operator as represented in (112).

**Table:** 

(112)	I_i_ had become adept at reading my teachers’ thoughts. [My Greek teacher]_k_ didn’t like me so much after all. [pro_i_-logFID [pro_k_-logFID *Really, I*_*i*_* was a little devil…*]]

In short, the outcome of this section is that several properties of FID (correct attribution of truth, ban on anti-attitudinal elements, *de se/de te* readings, sequence of tense phenomena, recursivity of FID) can be derived only if we posit an implicit attitudinal operator scoping over FID passages.

### A logophoric operator

This section specifies the nature of this attitudinal operator and argues that it must be a logophoric operator (log-FID) similar to logophoric operators independently motivated in the literature (see Koopman & Sportiche, 1989; Anand, 2006; Charnavel, 2019a, 2020, i.a.).

Logophoric operators have been hypothesized to explain the distribution of logophoric elements, such as specific pronouns mainly found in some West African languages (see Koopman & Sportiche, 1989, i.a.) and anaphors exempt from Condition A of Binding Theory (see Charnavel, 2019a, i.a.). As mentioned in Sect. 1.2.2, logophoric elements are characterized by having to refer to the perspective center of their domain, which constrains both their interpretation and their distribution.

Specifically, Charnavel (2019a, 2020), which I here adopt, posits that in a given domain, the relevant logophoric center can be syntactically represented by a silent logophoric pronoun *pro*_*log*_ introduced by a logophoric operator (as its subject), which imposes the first person perspective of the logophoric center on its complement. As logophoric centers encompass attitude holders and empathy loci, logophoric operators basically correspond to unspecified mental verbs imposing the perspective of the referent of their subject in their complement clause. A formalization is offered in (113).

**Table:** 

(113)	Semantics for the log-FID operator:
	for any world w, individual x, time t and function p from world-time-individual-assignment quadruples to truth values,〚 log-FID 〛 ^c, g^ (w)(p)(t)(x) = 1 iff for all world-time-individual-assignment quadruples <w′, t′, x′, g′> compatible with x′s mental state in w at t (where x′ is the individual in w’ that x identifies as himself), p(w′)(t′)(x′)(g′) = 1.

This explains the interpretation of logophoric elements (including attitudinal elements and *de se* pronouns discussed in Sect. 2.1). To account for the distribution of exempt anaphors, Charnavel further assumes that such anaphors are in fact not exempt from Condition A, but locally bound by the silent logophoric pronoun subject of the operator (see Sect. 1.2.2). This is why logophoricity and exemption correlate. Leaving empathy aside for now (see Sect. 2.4 for discussion), we can similarly hypothesize that FID passages are complements of a covert logophoric operator log-FID taking a silent subject *pro*, whose reference is determined on the basis of various discourse and syntactico-semantic factors. This explains why FID can contain exempt anaphors—if they refer to the FID protagonist—as in (50) represented in (114).

**Table:** 

(114)	[pro_i_-logFID [*That was one of the bonds between Sally and himself*_*i*_*.*]

Under the log-FID hypothesis, *himself* is here acceptable because it is locally bound by the subject *pro* of the log-FID operator (as required by Condition A; see some further discussion in Sect. 2.4). This is possible only because *himself* refers to the FID protagonist, to which the subject of the covert logophoric operator must refer. As detailed in Sect. 3, no existing analysis can otherwise derive this fact (although Sharvit’s analysis could easily be amended to account for it by assuming a syntactically represented subject for her FID operator).

Furthermore, Charnavel’s (2019a, 2020) logophoric operator forces *de dicto* readings in its scope to account for the perspectival homogeneity of logophoric domains (see, e.g., (93)). Assuming the presence of a log-FID scoping over FID passages thus accounts for why everything must be read *de dicto* in FID as discussed in Sects. 1.1.2 and 1.3.2 (cf. Sharvit, 2008,30).

**Table:** 

(115)	Obligatory *de dicto* under log-FID: local binding of all world variables
	pro-logFID-w_3_ [λw_6_ . . . XP- w_6_ . . .] is well-formed
	pro-logFID-w_3_ [λw_6_ . . . XP- w_(n≠6)_ . . .] is ill-formed

Recall that under DD-based accounts, this requirement is accounted for by quotation or internal context dependency; but under these accounts, mechanisms must also be designed to account for the anchoring of tense and person pronouns to the narrator (i.e., unquotation or external context dependency), which are partly at odds with the *de dicto* requirement (see Sect. 3).

There are two further properties that we saw can be descriptively classified under *de dicto* requirements in Sects. 1.2.1 and 1.2.2: the fact that pronominal gender and number judgments for individuals different from the protagonist must be attributed to the protagonist (see, e.g., (16)); the ban on *de re* bound reflexives in FID. Under DD-based accounts, this must be stipulated, as pronouns can generally depend on the external context. Under the log-FID hypothesis, however, this can follow from the independently motivated hypothesis that the operator imposes *de dicto* readings.^31^ More formally, I have adapted Sharvit’s (2008) hypothesis and assume that the logophoric operator quantifies over assignments (see (113)), and assignment functions in the scope of log-FID must be relativized to the protagonist’s belief.^32^

Given that a variable assignment reflects the beliefs of the speaker, the individuals in the range of the assignment must be acquainted with by the speaker. In the scope of log-FID that forces *de dicto* readings, pronouns thus refer to individuals as identified by the protagonist (unless they are lambda-bound and thus *de se* or *de te* pronouns as discussed in Sect. 2.1). Like Sharvit (2008), we can therefore conclude that their gender presuppositions must be relativized to the protagonist’s beliefs. In fact, note that it has been independently observed (by, e.g., Sudo 2012) that the gender presuppositions of pronouns in attitude contexts are filtered by the attitude verb if they are read *de dicto*. Further note that person presuppositions, however, are not relativizable due to their context dependency, as we will see in the next section (see (122)).

Similarly, the ban on *de re* bound reflexives in FID (see, e.g., (54)c represented in (117)) follows from the quantification of the log-FID operator on assignments functions.

**Table:** 

(116)	a.	〚She_i_ wanted PRO_i_ to hear herself_i_ sing again〛^g^	[✓*de re* interpretation]
	b.	#She_i_ wanted λg’. 〚PRO_i_ to hear herself_i_ sing again〛^g’^	[×*de re* interpretation]
	c.	*She_i_ wanted λg’. 〚PRO_i_ to hear herself_k_ sing again〛^g’^	[Condition A violated]
(117)	a.	*〚pro_i_ log-FID PRO_i_ to hear herself_i_ sing again〛^g^	[obligatory assignment binding violated]
	b.	#〚pro_i_ log-FID λg’. PRO_i_ to hear herself_i_ sing again〛^g’^	[×*de re* interpretation]
	c.	*〚pro_i_ log-FID λg’. PRO_i_ to hear herself_k_ sing again〛^g’^	[Condition A violated]

The ID in (54)b (as represented in (116)a) can be appropriate because the speaker may be responsible for the binding of *herself* by PRO (under matrix assignment g anchored to the speaker); the attitude holder, however, cannot, as represented in (116)b-c, because she is not aware of the coreference relation between herself and the woman on the radio: assignment g’ quantified over by the attitude verb induces either the wrong (*de se*) interpretation (under *de se* binding and feature deletion, as detailed in Sect. 2.1) or a violation of Condition A (cf. Sportiche, 2022). Given the hypothesis that the logophoric operator forces the assignment function to be anchored to the protagonist (i.e., the assignment function must be bound by log-FID, see (113) and fn. 33), we correctly predict that the FID in (54)c is unacceptable: the representation in (117)a is inappropriate because it violates this condition; the representation in (117)b, which induces a *de se* interpretation under assignment g’) does not induce the appropriate interpretation given that the protagonist is not aware of the coreference relation; and the representation in (117)c is unacceptable because it violates condition A (there is no coreference under assignment g’). Thus, the log-FID hypothesis allows us to further motivate Sharvit’s (2008) assumption that the FID operator quantifies over assignment functions; this property is not an idiosyncrasy of the FID operator, but derives from the property of logophoric operators to force *de dicto* readings generally, including with respect to (co)reference/valuation relations. Note that this relies on the idea that contrary to standard assumptions, (co)reference/valuation relations can be relativized to the attitude holder in attitude contexts in general, while they must in logophoric contexts (cf. Sportiche, 2022).^33^ Further note that this predicts that the referents of all pronouns (including first and second person pronouns, crucially) must be known to the protagonist (the FID logophoric center) to which the index is relativized. This is compatible with, and further specifies, the observations in Sect. 1.2.3, according to which the referents of indexical pronouns in communication FID contexts need not correspond to the protagonist herself or their addressee: they must, however, be known to the protagonist.

Finally note that the relativization of the assignment function to the logophoric center provides an explanation for the obligatoriness of *de se* readings observed in Sect. 1.2.1. If a pronoun referring to the protagonist is not *de se* bound by the feature deletion mechanism detailed in Sect. 2.1, its value must be assigned by the assignment function bound by log-FID, i.e., relativized to the protagonist. Assuming that the assignment function corresponds to referential intentions, it entails that a free pronoun could refer to the protagonist only if such reference corresponds to her intentions (not the narrator’s, crucially), which amounts to a *de se* reading. Does it mean that there are two routes for *de se* reading in FID? Taking into account the presuppositions of pronouns suggests that it is not the case: only *de se* binding is licensed. Indeed recall that the gender feature of a third person pronoun referring to the FID protagonist reflects the narrator’s judgment (see (16)a), not the protagonist’s. This only follows under *de se* binding. We can thus hypothesize that *de se* bound pronouns prevail over *de se* free pronouns due to Rule I (Reinhart, 1983, i.a.).^34^

In sum, the log-FID hypothesis allows us to derive simultaneously a set of properties that must be stipulated or remain unexplained under DD-based analyses using a mechanism (the logophoric operator) that is independently motivated. In other words, the hypothesis that FID is a kind of logophoric context explains why in FID, we observe an array of properties observed in logophoric contexts in general such as obligatory *de se* and *de dicto* readings. At the same time, it also accounts for why this set of properties is associated with another set of properties (anchoring of tense and person to the speaker), which are also attested in other logophoric contexts. On the contrary, this association is problematic under DD-based analyses.

Furthermore, the log-FID hypothesis makes predictions that seem to be borne out in the case of recursive embedding. Charnavel (2019a, 2020) argues that logophoric domains must be perspectivally homogeneous, in the sense that the whole domain must be evaluated by the same logophoric center (see (93)). This predicts that in the case of FID double embedding, all elements must be anchored to the same protagonist. An example provided by Eckardt (2014, p. 58) confirms the prediction:

**Table:** 

(118)	Little Mr. Bowley pushed his way through the crowd. *Where for God’s sake did all these unfortunate creatures come from?* (Eckardt, 2014, p. 58, adapted from Woolf, *Mrs. Dalloway*)

Under the recursive FID reading where (118) is part of Mrs. Dalloway’s train of thought, the underlined evaluative expressions crucially give rise to only two interpretations: either both are anchored to Little Mr. Bowley, or they are both anchored to Mrs. Dalloway. A perspectival mix, where Mrs. Dalloway is responsible for the question, but Mr. Bowley is for the qualification *unfortunate*, or vice versa, is unavailable, according to Eckardt. This is predicted by our hypothesis:

**Table:** 

(119)	[pro_i_-logFID [pro_k_-logFID [Where for God’s sake did all these unfortunate creatures come from?]]]

Under the log-FID hypothesis, the FID in (118) is out-scoped by two logophoric operators taking Mrs. Dalloway and Mr. Bowley for subjects, respectively. As independently argued by Charnavel (2019a), the perspective of a logophoric domain out-scoped by two different logophoric operators can be set to either perspective; however, it must crucially be perspectivally homogenous.^35^ Thus, *where for God’s sake* and *unfortunate* must both depend on the same operator, either the one expressing Mrs. Dalloway’s perspective, or the one expressing Mr. Bowley’s, but no mix is possible: only one operator can be active in a given domain.

In sum, the log-FID operator is a specific kind of attitudinal operator that imposes universal and homogeneous *de dicto* and *de se* readings in its scope, that is, it forces anchoring of all variables (pronouns, times, worlds) as well as the assignment function to the (same) protagonist. Crucially, this kind of operator is independently motivated for logophoric contexts, and need not be assumed to be specifically designed for FID as is the case of Sharvit’s (2008) operator, which achieves most of the same effects.

### Deriving the behavior of indexicals in FID

Another conspicuous difference with Sharvit’s (2008) FID operator is that the log-FID operator does not quantify over contexts. Specifically, Sharvit’s operator quantifies over context-assignment pairs. Furthermore, Sharvit adapts dual context dependency to her analysis to account for the difference between first and second person pronouns, which do not shift in FID, and adverbial indexicals, which always do according to Sharvit and most of the previous literature. More precisely, the lexical entry of first and second person pronouns specifies that they must depend on the external context of evaluation, while adverbial indexicals like *tomorrow* depend on the internal context whenever it is available.^36^ This aspect of Sharvit’s account, which makes it hybrid (see Eckardt, 2014, p. 194), seems to be the reason for its unpopularity (see criticisms in Eckardt, 2014; Maier, 2015; Reboul et al., 2016, i.a.), even if it is the only existing account able to derive (vs. stipulate) *de se* (and *de te*) readings, sequence of tense phenomena and gender/number pronominal judgments. The present account aims at improving on Sharvit’s account, not only by independently motivating the FID operator (as a specific kind of logophoric operator), but also by abandoning dual context dependency and quantification over contexts. In fact, our new observations in Sect. 1.3.2 support the idea that the flexibility of adverbial indexicals is not due to FID itself, but to narrative (vs. communicative) discourses, which thus justifies attributing their flexibility not to FID itself, but to the pragmatic specificity of narration or fiction.

The main, crucial argument for this move is the new observation detailed in Sect. 1.3.2 that, contrary to the predictions of all previous proposals, time and location indexicals in FID must be anchored to the narrator when the narrator is a regular speaker involved in a conversation with a specific addressee at a specific time and location, as in (102) repeated below in English.

**Table:** 

(120)	X called me three days ago to explain the reasons for his absence: he had {*yesterday/ the day before} contracted a bad cold.

*Yesterday* is here unacceptable because it must be anchored to the speaker’s (vs. X’s) time, thus making the discourse incoherent. This directly follows from the log-FID hypothesis, given that indexicals do not shift under logophoric operators in general (see Charnavel, 2020, p. 698, fn. 34). However, *yesterday* is incorrectly predicted to be relativizable to X’s time under Sharvit’s account (due to quantification over contexts by the FID operator and internal context dependency of *yesterday*) as well as under DD-based analyses.

Similarly, first and second person pronouns are correctly predicted to refer to participants of the actual context of utterance under the log-FID hypothesis without having to stipulate external context dependency in their lexical entries.

**Table:** 

(121)	X [m’_i_]a appelé. *Est-ce qu’on peut l’emmener, toi*_*k*_* ou moi*_*i*_*, à Roissy, à 5 heures du matin, demain ?* Il rêve ! [oral 10–04–2005] (Authier-Revuz, 2020, p. 314)
	X called [me_i_]. *Can we drive him, you*_*k*_* or me*_*i*_*, to Roissy, at 5am tomorrow?* He is dreaming!’

In (2) repeated above, *toi* thus refers to the addressee of the actual speaker, not to X’s addressee (which is the speaker and is thus referred to by *moi*), because the indexical presupposition associated with *toi* is relativized to the actual context of utterance.

Note that similarly, *moi* refers to the actual speaker, not to X. But since the speaker here corresponds to X’s addressee, this is not derived by the same mechanism, but from *de te* binding and feature deletion discussed in Sect. 2.1 and fn. 30 (cf. Sharvit, 2008; Cumming & Sharvit 2016) as schematized in (122) (simplified for readability as to express *can you or me drive him to Roissy?*).

**Table:** 

(122)	

Thus, first and second person pronouns are interpreted in FID as indexicals depending on the actual utterance only when they are not read *de se* or *de te* (which, as we saw in Sect. 1.2.3, only happens when FID occurs in communication contexts). Further note that the behavior of person indexicals does not contradict the universal *de dicto* requirement of our log-FID operator. We argued in Sect. 2.2 that assignments of pronoun references must be relativized to the protagonist in FID. This (correctly) entails that in (121)-(122) above, it is X that is responsible for the reference of *toi* (i.e., g’(k)). But this is independent of the indexical computation (i.e., the reference of a_c_, say Tim) that is part of the person presupposition and is not relativizable: X is only responsible for identifying g’(k) with Tim. Because of their context (vs. world) dependency, person presuppositions thus behave differently from gender (and number) presuppositions in this respect (see Sect. 2.2).

Another argument for abandoning dual context dependency is the other new observation in Sect. 1.3.2 that anaphoric (vs. indexical) adverbials are also felicitous in FID, as exemplified in (100) partially repeated below.

**Table:** 

(123)	*Voyons, ce serait pour le lendemain dix heures*.
	‘Come on! it would be at ten o’clock the next day.’

The temporal anaphoric *le lendemain* ‘the next day’ is straightforwardly predicted to be acceptable under ID-based analyses (just as it is in ID), but requires at best some stipulations under DD-based analyses (see details in Sect. 3). More specifically, we can treat time anaphoric adverbials as containing a time variable (e.g*., the day after t*). When the variable is free, it can be evaluated with respect to any salient time in the discourse, thus explaining anaphoric uses in general. In FID, temporal anaphoric adverbials can have such anaphoric uses, which are expected under our hypothesis that the log-FID operator forces *de dicto* readings: the protagonist is responsible for both the evaluation of the definite description and for the value assignment of the time variable. That said, (123) exhibits a special case where the time variable denotes the actual time of the protagonist. Such use is precluded in DD, where the use of a counterpart indexical like *demain* (where the time variable is specified as being indexical) prevails. In FID, this is however licensed under our log-FID hypothesis because the time variable can be bound by the time coordinate of the operator (*de nunc* reading).^37^

The explanatory burden for our log-FID hypothesis is to account for the well-known observation—often considered to be a hallmark of FID and motivating bicontextual dependency in most previous analyses—that adverbial indexicals are anchored to the protagonist in narrative FID as discussed in Sect. 1.1.2 and illustrated in (14), repeated below in English.

**Table:** 

(124)	*Down his horse, he was walking […] and he was smiling, strangely and princely, certain of a victory. Twice, yesterday and the day before, he had been a coward, he hadn’t dared. Today, on this first-of-May day, he would dare and she would love him*.

Here, all adverbial indexicals are clearly anchored to the protagonist (Solal). But given that our log-FID operator does not quantify over contexts, they should depend on the actual utterance, namely the narrator’s.

To account for this fact, I would like to appeal to the specificities of the narrative context in which the FID is inserted. (124) is the incipit of Cohen’s novel *Belle du Seigneur*. As often happens in fiction, no clue is given about the narrator and her circumstances, so that the context of narration remains unspecified (i.e., it is a set of possible contexts, as discussed in Eckardt, 2020, 2021). The intuitive idea I would like to pursue here is that in the absence of a well-defined actual context of utterance, the reader interprets the indexicals with respect to a context that is fully identifiable and salient, namely that of the protagonist. In a nutshell, I will assume that indexicals are always interpreted with respect to the matrix context, but the matrix context can be an improper one under some pragmatic conditions, i.e., if the actual context of utterance is unspecified or irrelevant (as is usually the case in fiction). Furthermore, I will make a distinction between simultaneous adverbials like *now/today* and non-simultaneous adverbials like *yesterday/tomorrow*: due to their reflective meaning, the latter must occur in a speech act, which constrains the improper context to contain a specific author (and thus bans them from authorless narration without ID or FID).

More precisely, some narrative contexts (esp. fictive ones) can be treated as improper contexts, i.e., as intended contexts of interpretation as in Predelli (1998a, 1998b). Predelli (1998a, 1998b) argues that in cases where the addressee is not in presence of the speaker, but receives the message later, the relevant context for interpreting indexicals need not be the actual context of utterance, but can be a context in which the speaker intends the addressee to interpret the message (henceforth, the intended context of interpretation). For instance, *I am not here now* heard from a recording machine is not incoherent because the context in which the speaker intends the sentence to be interpreted is not the context in which she actually uttered the sentence, but the context in which she intends the sentence to be heard. To be more concrete, imagine that I wrote a note when I was at home saying *I am not here now* and later put it on my office’s door just before going on holidays. The note is interpretable if the adverbial indexicals are relativized to the intended context of interpretation (i.e., the office during the holidays), not the actual context of utterance (i.e., home before the holidays): *I* refers to the author of the message (even if she wrote the note in the past, before the message is decoded), but *here* denotes the location of the intended context of decoding (i.e., the office) and *now* the time of the intended context of decoding (i.e., any time during the holidays). For the message to be felicitous, the reader of the note must recognize and accept that the note has been intended to be interpreted at those time and place. In other words, Predelli assumes the existence of improper contexts, in which it may not be the case that the time coordinate is the actual time of utterance and the location coordinate the actual location of utterance.

Similarly, we can treat (some) narrative contexts as improper contexts, i.e., assume that in (some) stories, indexicals can also be interpreted with respect to an intended context of utterance. But in this case, the relevant coordinates are not those of the intended addressee (who can be any reader situated in any time and space), but those of the story in which the writer wants the reader to be absorbed.^38^ Predelli (1998a) explicitly makes this hypothesis and illustrates it with (125)-(126).

**Table:** 

(125)	It is 1796. Napoleon, now commander of the French troops defeats the Sardinian forces and turns against Austria. (Predelli, 1998a, p. 112)
(126)	Here, to the sheltered columned coolness, Ramanujan would come. Here, away from the family protected from the high hot sun outside, he would sometimes fall asleep ....
	(Predelli, 1998a, p. 113 from Kanigel, 1991, p. 29-30)

We observe that (125) involves the historical present, which may be argued to involve some kind of shifting or bicontextual dependency (see Schlenker, 2004; Anand & Toosarvandani, 2017, i.a.), but crucially, the presence of the historical present is not necessary to license the indexical *now* here as observed by Recanati (2004): see (94). Moreover, both examples include *now* and *here*, which we mentioned in Sect. 1.3.2 are (like *today*) usually recognized to be more flexible than other adverbial indexicals such as *tomorrow* or *yesterday* (see Kamp & Reyle, 1993; Recanati, 2004; Hunter, 2012; Eckardt, 2014; Altshuler, 2016; Lee, 2017; Anand & Toosarvandani, 2019, i.a.). While *now/today/here* exhibit non-indexical uses outside FID (as in (125)–(126)), the non- (or shifted) indexical uses of other adverbials like *yesterday* or *tomorrow* are confined to FID (and ID, see Sect. 1.3.2).

To account for the difference,^39^ I propose combining Predelli’s approach with Abrusán’s (2021, p. 859) hypothesis that adverbs like *yesterday* come with a lexical presupposition that makes their interpretation contingent on being used in a speech act. As we will see in Sect. 2.4, Abrusán’s hypothesis is motivated by the different behavior of these indexicals in FID (expressing a protagonist’s thought or speech) and in PP (expressing a protagonist’s nonverbal perception). Here, I more specifically assume that the use of non-simultaneous temporal adverbs such as *yesterday* (vs. simultaneous adverbs like *now*) presupposes a reflective mental state that is linguistically explicit as exemplified in (127).

**Table:** 

(127)	〚*yesterday*〛^g, c^= the day before t_c_
	= # iff it does not occur as part of a speech act by s_c_. (cf. Abrusán’s, 2021, p. 859)

The intuition behind this hypothesis is that only the expression of non-simultaneity requires a conscious and linguistic computation by some mental agent. Thus, indexicals expressing simultaneity like *now* can be used (in a way similar to anaphoric adverbials) in narrative discourses such as (125)-(126), which do not involve any salient author of a linguistic act (the narrator of the story being not identified as sufficiently relevant or salient). But non-simultaneous adverbs like *yesterday* or *tomorrow* in narratorless fiction can only occur within discourses like FID (or ID^40^) expressing a speech or thought act by some agent (the protagonist), which can satisfy their lexical presupposition due to improper contexts that can identify the author with the protagonist. The three main relevant behaviors of adverbial indexicals are summarized in Table 2.

**Table 2:** Main behavior of adverbial indexicals in narration

	FID (or any other discourse) in specified context of narration (communication)	FID (or ID) in unspecified context of narration (story)	Non-attitudinal discourse in unspecified context of narration (story)
Indexical adverbials expressing simultaneity e.g., *now*	Anchored to narrator (or protagonist) e.g., (101)	Anchored to protagonist e.g., (14)	Anchored to protagonist e.g., (125)-(126)
Indexical adverbials expressing non-simultaneity e.g., *tomorrow*	Anchored to narrator e.g., (102), (106)	Anchored to protagonist e.g., (14), (98)	Infelicitous e.g., (142)

To sum up, the behavior of non-simultaneous adverbial indexicals directly derives from the combination of Predelli’s and Abrusán’s hypotheses. Due to their lexical presupposition, they must be used as part of a reflective, linguistic act. Due to their indexicality, the author of this act is by default the context author, i.e., the actual speaker or narrator (as in communication). But in the absence of a speaker (or salient narrator) at the intended time of interpretation as in fiction, the anchor can be a protagonist—due to the availability of improper contexts—if this protagonist is the author of a linguistic act (as in FID). Alternative analyses attributing to FID their shiftability (due to the FID operator or bicontextual dependency) cannot, however, derive the specificity of their behavior, since crucially, FID is neither a necessary condition for them to shift (see (98) involving ID), nor a sufficient one (see (102), (104) without a shift in communicative context). As for simultaneous adverbials, they are more flexible because their interpretation is not contingent on being used as part of a linguistic act (and potentially also for other reasons to be further explored; see fn. 40).

This account thus seems to rely on a distinction between cases where the context of narration is specified (as in FID in communication) and cases where it is not specified (as in FID in narratorless fiction). But as noted by an anonymous reviewer, intermediate cases of specification such as (128) require further explanation.

**Table:** 

(128)	a.	Two weeks ago, on June 28, 2022, Mary was walking home from school. *Hooray, {today /?tomorrow} was her last day of classes*, she thought.
	b.	〚 *tomorrow*〛 ^g, c^ = the day after t_c_ [= June 29]
		= # iff it does not occur as part of a speech act by s_c_ [s_c_ = Mary]

Here, the context of narration is only specified by the indexical *two weeks ago*. In this case, the simultaneous indexical *today* seems acceptable, and the non-simultaneous indexical *tomorrow* seems to have an intermediate status. As mentioned, simultaneous indexicals like *now* or *today*, which need not be interpreted as part of a linguistic act, are more flexible (thus requiring further research; see fn. 40), so the case involving *tomorrow* is here most problematic: why can *tomorrow* be (at least marginally) anchored to the time of the protagonist, while the time of narration is specified (by *two weeks ago*)? Under our hypothesis, this amounts to specifying the conditions under which the context of the story (vs. narration) can be treated as the intended context of interpretation: in (128), under what conditions can the matrix context be resolved as Mary’s context?

First, we observe that the use of *tomorrow* becomes unacceptable if the discourse is further specified as FID inserted in an actual conversation where not only the time, but also the speaker and the addressee are specified.

**Table:** 

(129)	a.	Leo tells Julie on July 12: “You know, I am convinced that Mary does not like school. Two weeks ago, on June 28, I was walking home with her from school. Hooray, {*tomorrow/the next day} was her last day of classes, she told me.”
	b.	〚*tomorrow*〛^g, c^= the day after t_c_ [= July 13]
		= # iff it does not occur as part of a speech act by s_c_ [s_c_ = Leo]

This confirms that the context of the story cannot be construed as the intended context of interpretation if the context of the actual speaker is fully specified (see (102), (104)). In particular, we saw that improper contexts are usually accessible when the addressee is not in presence of the speaker, but receives the message later.

Second, I assume that *tomorrow* in (128) can be accepted only to the extent that the context of narration can be construed as sufficiently irrelevant or non-salient, which depends on its context of insertion, esp. whether it is part of a dialogue with a specific addressee (as in (129), in which case *tomorrow* cannot shift) or part of a story with no specific addressee, in which case shift can happen (as in (128)). In the latter case, the story certainly starts with an anchoring in the actual context, but soon, the relevance of this context fades away in the absence of further specification about the narrator (who does not take part in the story) and her addressee. The reader can thus reset the context of interpretation as that of the story and interpret t_c_ as June 28 (note that a radical instance of such context move routinely occurs after incipits involving expressions such as *a long time ago*).

In sum, it is not the underspecification of the context of narration per se that licenses the construal of the story coordinates as the intended context of interpretation, but its irrelevance for interpretation (as is also the case of Predelli’s examples that do not involve fiction). The irrelevance of the context of narration directly obtains when it is not specified at all (esp. in narratorless fiction), but it can also obtain when it is partially specified (as in 128) under certain pragmatic conditions (e.g., narrator not involved in story, absence of specific addressee). These conditions remain to be further clarified in future research; for our purposes, it is sufficient to observe that it is the remoteness of the narrator’s context (however this notion can be further specified), and not FID per se, that allows the anchoring of adverbial indexicals to the protagonist’s parameters.^41^

Finally note that so far, we have examined examples of improper contexts where time and location (as in, e.g., *yesterday* or *here*) are not the coordinates of the actual context of utterance (although the lexical presupposition of *yesterday* also involves the author coordinate). What about the author and addressee coordinates? Regarding the author first, it seems that the improper context hypothesis, unlike bicontextual accounts, implies that it need not refer to the actual utterer. In fact, Predelli (1998b) provides an example of improper context in which the agent coordinate is not the actual author of the utterance in (130). The sentence is uttered by a lecturer commenting on the *Nicomachean Ethics* in an introductory class.

**Table:** 

(130)	I argued at length that one lives the best life by exercising both moral and intellectual virtues. And now I am suddenly advocating a rather different position, namely that the good life must be devoted solely to theoretical activity. Do you see a way out of this apparent inconsistency?

Here, the first person pronoun is used to refer to Aristotle rather than the lecturer, who is temporarily pretending to be in Aristotle’s shoes. Is this case attested in FID? At first glance, this does not seem to be the case since first person pronouns cannot refer to a protagonist distinct from the narrator; we thus claimed that their interpretation must depend on the utterance context (see, e.g., Sect. 1.2.1.). But crucially, this claim relies on the assumption that the author of the utterance context is the narrator. As often acknowledged (see, e.g., Abrusán, 2021, p. 861, fn. 29), this assumption is too simplistic, and glosses over the difference between author and narrator. In the sense of Kaplan, the author of the actual context of utterance must be the actual author of the fiction; the narrator is not a proper context agent since a narrator is not situated in a specific context of utterance: her context must rather be conceived as a set of possible contexts (cf. Eckardt, 2020, 2021; Abrusán, 2021, p. 855, fn. 23). It seems to me that this tension can be resolved if we hypothesize that the narrator is the author of an intended context of interpretation in Predelli’s sense. Under this hypothesis, the author in (130) corresponds to the lecturer, and the narrator to Aristotle.

First-person fictive narrations can thus be treated as involving an improper context where the author (the narrator) corresponds to a coordinate of an intended parameter of interpretation. This makes a prediction to be tested in future research: first-person fictive narrations should presumably disallow adverbial indexicals within a FID anchored to a third person protagonist (unless improper contexts can vary in the course of a narration). This situation would indeed create a conflictual requirement to resolve the value of the author coordinate of the improper context: should it be the narrator (given the first-person narration) or the third person protagonist (given the presupposition of the adverbial)?^42^

As for the addressee coordinate, our hypothesis similarly predicts a second person pronoun to be able to refer to the addressee of an intended context of interpretation in the absence of an actual addressee. Predelli (1998a) provides an example of intended addressees in (131):

**Table:** 

(131)	[recording machine] You have reached 123-4567. Please leave a message after the beep.

*You* here does not refer to the actual addressee of the recorder of the message (there was probably none), but to the intended addressee of the recorded message (any potential caller). In fiction, this case corresponds to cases where the (individual) reader is referred to by *you*. The case is quite rare as it implies that the intended reader is involved in the narrative, which puts strong constraints on the narrative and creates specific effects since the intended reader thereby becomes more specified—through details of the story—than the actual reader, which can be anybody (unlike the narrator, which is never fully specified in a story and is thus conversely less specified than the actual author). This has been exploited in second-person narrations (famously in, e.g., Butor’s *Second thoughts*—French* La Modification*). Furthermore, although all our examples of Sect. 1.2.1 with second person pronouns exhibit FID in communication, FID can also occur in second-person narratives, as observed by Fludernik (1993, pp. 81–82) with (132).

**Table:** 

(132)	Sunlight. A morning. *Where the hell are your sunglasses?* You hate mornings—anger rises in you, bubbling like something sour in your throat—but you grin into the morning because somebody is approaching you, shouting a magic word. Your name. [‘*You’*, Oates] (Fludernik, 1993, p. 82)

*You* here refers to the protagonist and is thus a *de se* pronoun whose person features are not interpreted. As in the case of first person pronouns in FID in first-person narration, the context of the person feature is therefore not determined by the FID context but by the intended context of the story. This confirms that the apparent shiftability of some indexicals in FID does not derive from FID itself, as correctly predicted by our log-FID operator hypothesis.

Finally note that if our hypothesis correctly licenses some uses where first and second person pronouns do not refer to the actual speaker or addressee, it does not overgenerate uses where they would incorrectly refer to an FID protagonist or addressee. As reviewed in Sect. 1.2.1, recall that third person protagonists are referred to by third person pronouns in FID. Why can’t they alternatively be referred to by first person pronouns interpreted as depending on an improper context? This is because, as mentioned in Sect. 2.2 (see also fn. 35), pronouns referring to the protagonist (or her addressee) must be *de se* (or *de te*) bound. This hypothesis correctly predicts that protagonists (or addressees) in FID can only be referred to by first (or second) person pronouns in FID if they are also denoted by first (or second) person pronouns outside FID (i.e., in first or second person narratives or in communication contexts).

### Crosslinguistic implications and open issues

In sum, I have argued that our log-FID hypothesis coupled with the improper context hypothesis can correctly predict the empirical properties of FID in languages like English, French, German, Italian or Hebrew. Furthermore, it makes specific predictions for other languages. First, it predicts that in languages with logophoric pronouns, the protagonist in FID can (or perhaps should) be referred to by a logophoric pronoun (contrary to Schlenker’s explicit predictions, see fn. 48). This seems to be borne out, at least in Ewe (but see Maier, 2024 for a different view). The literature on logophoric pronouns does not mention FID, but reports instances of discourses where the logophoric pronoun occurs in unembedded sentences after an attitude report has been introduced (see Clements, 1975; Koopman & Sportiche, 1989; Adesola, 2005; Pearson, 2015, i.a.). This is illustrated in (133)-(134) in Ewe.

**Table:** 

(133)	Kofi	koudrin	be	yè	bidzi.	*Marie*	*zu*	*yè*.
	Kofi	dream	compl	log	angry	Mary	insult	log
	‘Kofi_i_ dreamed that he_i_ was angry. *Mary insulted him*_*i*_.’ (Pearson, 2015, p. 96)							


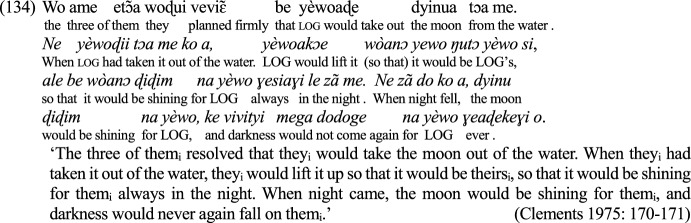
The second sentence in (133) is not syntactically embedded under the attitude verb *dream* and the complementizer *be*. But as noted by Pearson, it is semantically embedded, as it implies that the insult took place in the dream.^43^ This seems to correspond to FID, and crucially, the logophoric pronoun *yè* is used to refer to the dreamer. (134) is an excerpt from an oral retelling of the tale “the Monkeys and the Moon”. Again, the logophoric pronoun referring to the protagonist (the three monkeys) occurs throughout the discourse, even if only the first sentence includes an attitude verb; furthermore, the whole discourse is understood under the scope of this attitude verb. I leave for further investigation a more careful examination of FID in languages with logophoric pronouns, but these examples are promising with respect to our hypothesis.

Second, the log-FID hypothesis makes a clear prediction for languages with indexical shift, as mentioned by Sharvit (2008, pp. 358–359). Given that the log-FID hypothesis (like Sharvit’s hypothesis) likens FID to ID, it predicts that person indexicals should be shiftable in FID, i.e., the protagonist could (or should, depending on the shifting properties of the language) be referred to using a first person pronoun (and her addressee a second person pronoun). The relevant data are not accessible yet, but future research should determine whether the prediction is borne out.

I also leave for further research the investigation of some issues that remain open under this (and other) proposal(s). First, the constraints on the distribution of parentheticals in FID remain to be specified. As noted by Sharvit (2008, pp. 392–393), parentheticals do not license negation (e.g., (135)), license *only* only in certain conditions (e.g., (136)a vs. (136)b), but license modals (e.g., (137)). In those respects, FID contrasts with ID and DD, which both license all these options.

**Table:** 

(135)	a.	#*She was pregnant*, Mary didn’t say.
	b.	#*She was pregnant*. Mary didn’t say that.
(136)	a.	#*She was pregnant*, only Mary said.
	b.	*She was pregnant*. Only Mary said that.
(137)	a.	*Yes, she was pregnant*, Mary may have said.
	b.	*Yes, she was pregnant*. Mary may have said that.

As noted by Giorgi (2010, pp. 205–209), the parenthetical further obeys some constraints of insertion: generally, it occurs in post-sentential position or after a topic (i.e., as given information with respect to the protagonist’s context).

**Table:** 

(138)	*Sarebbe partita domani*, pensò.
	‘She would leave tomorrow, she thought.’ (Giorgi, 2010, p. 205)
(139)	*Domani*, pensò, *Gianni sarebbe partito*.
	‘Tomorrow, she thought, Gianni would leave.’ (Giorgi, 2010, p. 206)

However, it seems that parentheticals in FID are not semantically constrained: as observed by Banfield (1982, Ch. 2, Sect. 2), they can express any mental attitude (including any kind of speech or thought) and we find verbs of consciousness like *deplore* or *ponder* that can appear with FID, but not with DD or ID. This is correctly predicted by our hypothesis treating the logophoric operator as an unspecified mental verb that only semantically (vs. syntactically) embeds an attitude. The role of the parenthetical, then, is to specify the kind of mental attitude that is reported in FID.

Second, our hypothesis crucially relies on the idea that the same kind of operator is used in FID and in logophoric contexts, thus making FID a kind of logophoric context. But besides the differences due to syntactic (un)embedding (including the (im)possible presence of a parenthetical), which I have argued are independent of the presence of the operator, there seems to be a difference pertaining to the size of the logophoric domain. While logophoric operators are often assumed to scope over the whole proposition (they take a CP as argument, see, e.g., Koopman & Sportiche, 1989; Anand, 2006), Charnavel (2019a, 2020), which I here adopt, argues that logophoric domains can be smaller than CP, and include different types of spell-out domains (TP or any other XP with subject). This explains why apparently exempt anaphors are licensed in logophoric contexts: they are locally bound by the subject of the logophoric operator, which is contained in the same spell-out domain (which Charnavel argues is the relevant locality domain for Condition A; see Charnavel & Sportiche, 2016). The question raised by the extension of this hypothesis to FID bears on the fact that FID seems to systematically consist of full sentences.

Under the logophoric operator hypothesis, we can assume that the log-FID operator usually sits above TP, and that—as Charnavel assumes—co-valued logophoric operators can appear in the smaller domains of the same sentence (as can be revealed, e.g., if an exempt anaphor is contained within a DP with subject). But the question arises as to why we do not seem to find instances of FID covering smaller domains than full sentences. One approach to address this question is to assume that we do in fact observe such instances. Charnavel (2020, p. 688) in fact suggests that the logophoric contexts she examines could be conceived as embedded FID. One potential problem with this hypothesis is that adverbial indexicals are evaluated with respect to the speaker’s context in these smaller logophoric contexts, while they can be evaluated with respect to the protagonist’s context in FID as we discussed at length. But the solution we proposed for explaining this fact in FID (the use of improper contexts) also provides a solution for this apparent problem, as an improper context must be relevant for a full proposition. Another, compatible, approach to address the question is to derive the difference in domain size between FID and other logophoric contexts from the syntactic difference between them: as we saw, FID is syntactically unembedded, while logophoric ID is syntactically embedded. Positing a log-FID operator within a smaller domain (say, a DP) within an unembedded sentence would imply that this domain is uniformly evaluated by the protagonist while the rest of the sentence is uniformly evaluated by the speaker. This is not impossible (and some instances of mixed quotation could be reexamined under that light;^44^ see also examples in Harris & Potts, 2009), but imposes strong pragmatic conditions that could explain their rarity.

Finally, the log-FID hypothesis raises a question regarding the apparent restriction of FID to attitude contexts. FID standardly expresses represented speech or thought and thus corresponds to attitude contexts. Although logophoricity is sometimes assumed to also be restricted to attitude contexts (see, e.g., Schlenker, 1999), Charnavel (2019a, 2020) argues that it encompasses all types of mental perspective, including some non-attitude contexts (cf. Oshima, 2006; Sells, 1987, i.a.). Specifically, Charnavel proposes that logophoric contexts can be divided into two types of perspectival contexts, attitude and empathy, and the logophoric operator is unspecified with respect to the type of perspective it encodes, which is determined by a mixture of syntactic, semantic and pragmatic factors. Under the log-FID hypothesis, we thus expect to find instances of FID that correspond to empathy contexts. In the rest of this section, I want to suggest that this is indeed the case, and these instances have received different names such as PP (see (3)).

Inspired by the literature on Japanese (see esp. Kuroda, 1973; Kuno & Kaburaki, 1977), Charnavel (2019a, 2020) defines an empathy locus as a perceptual center of perspective, i.e., an event participant that the speaker identifies with from a sensory or emotional perspective: according to Charnavel (2019a, p. 166), “under empathic conditions, the speaker puts herself in the empathy locus’s shoes to report his first-personal perception”. While attitude contexts report beliefs subject to errors, empathy contexts report (potentially unconscious) experience immune to errors. Empathy loci can be diagnosed by specific lexical items, such as Japanese sensation adjectives (such as ‘be cold’, which express an inherently first-personal sensation; see Kuroda, 1973), Japanese giving verbs (implying that the event is described as experienced by the giver or by the receiver or from the outside, depending on the choice of verb; see, e.g., Kuno & Kaburaki, 1977) or *son cher* in French (‘her dear’, expressing an evaluation that can only be made by the individual experiencing the feeling; see Charnavel, 2019a, 2020). Thus, Charnavel (2019a, 2020) argues that exempt anaphors like *son propre* or *lui-même* in (140)a-b are licensed in spite of occurring in non-attitude contexts, because they are in empathy contexts, as can be diagnosed by the acceptability of co-referring *son cher*: they can thus be locally bound by the empathy center (represented by a logophoric pronoun subject of a logophoric operator).

**Table:** 

(140)	a.	Le courage de Paul_i_ a [_VP_ pro_log-i_ sauvé des flammes {sa_i_ propre /sa_i_ chère} maison et celle de ses voisins].
		‘Paul_i_’s courage [_VP_ pro_log-i_ saved from the fire {his_i_ own/his_i_ dear} house and his neighbors’].’
	b.	Emile_i_ mérite que Sophie pense [_VP_ pro_log-i_ à {lui_i_-même/sa_i_ chère fille} et à sa famille.]
		‘Emile_i_ deserves Sophie [_VP_ pro_log-i_ thinking about {himself_i_ /his dear daughter} and his family].’

Empathy as defined by Charnavel (2019a, 2020) bears a striking resemblance to the lesser known cousin of FID described under the names of represented perception (Brinton, 1980), non-reflective consciousness (Banfield, 1982; Kuroda, 1973), narrative perception (Fludernik, 1993), viewpoint shift (Hinterwimmer, 2017), or PP, which I will henceforth adopt (Abrusán, 2021; Holton, 1997; Stokke, 2013). As summarized by Abrusán (2021), examples of PP such as (3) or (141) describe the non-reflective mental states of a protagonist such as perceptions, emotions or nonverbal beliefs.

**Table:** 

(141)	When Mary stepped out of the boat, *the ground was shaking beneath her feet* for a couple of seconds. (Abrusán, 2021, p. 845)

Rather than describing a conscious thought, the italicized passage in (141) seems to capture a perception or sensation as experienced by Mary, and that in (3) represents the perception of the character as she looks out the window of the train. According to Abrusán (2021), PP, like FID, is an instance of a more general category of perspective shift. The differences between PP and FID are the following: PP is not a report of thoughts or speech, but a report of pre-linguistic mental states; PP, unlike FID, can be embedded (see (141)); PP does not license the evaluation of adverbial indexicals with respect to the protagonist’s context (see (142)).

**Table:** 

(142)	A week ago, Ann was pacing around after coming home from the jeweller, disappointed and angry with John. *{#Yesterday/the day before} he gave her a ring studded with diamonds*, but they turned out to be glass. (Abrusán, 2021, p. 858, citing Stokke, 2013)

According to Abrusán (2021), the first two properties derive from the pragmatic licensing of PP realized by rules yielding an update of the story with what the character feels and perceives (vs. thinks or says in FID); and speech or thought acts are root-level phenomena. As mentioned in Sect. 2.3, the latter property, she argues, comes from a lexical presupposition of (non-simultaneous) adverbial indexicals that require them to be used in a speech act: in other words, the use of temporal adverbs such as *yesterday* presupposes a reflective mental state that is linguistically explicit.

Although Abrusán couches her analysis in terms of Eckardt’s bicontextual account, her observations can directly be captured by our logophoric approach: PP corresponds to empathy, and perspective shift to logophoricity. PP and FID can thus be uniformly captured by the log-FID hypothesis, which relies on the presence of an unspecified logophoric operator. As in Abrusán (2021), the difference between empathy (PP) and attitude (FID) comes from a combination of syntactic, semantic and pragmatic factors that determine the type of perspective realized by the operator. As in Abrusán (2021), and as suggested above, the difference in domain size comes from the syntactico-pragmatic constraints imposed by the type of perspective. Finally, Abrusán’s (2021) explanation regarding the unshiftability of adverbial indexicals in PP is compatible with our logophoric account: as discussed in Sect. 2.3, the log-FID operator does not yield context shift; the appearance of shift in some cases merely comes from the availability of improper contexts in some narrative contexts, which happen to license FID. If we assume, like Abrusán (2021), that non-simultaneous adverbial indexicals require linguistic consciousness, it follows that they cannot be evaluated with respect to an improper context corresponding to the protagonist’s context in PP. In fact, we independently know that non-simultaneous adverbial indexicals do not shift in empathy contexts.

In sum, the existence of PP provides an additional argument for our log-FID hypothesis, as it instantiates the empathy counterpart of FID, which is expected to be attested given that under the hypothesis adopted here, logophoricity encompasses both empathy and attitude.

## Problems with previous analyses

In Sect. 2, we have seen that a logophoric, ID-based analysis can capture all empirical properties of FID presented in Sect. 1, thus faring better than DD-based analyses. To make this point more precise, this last section is devoted to reviewing previous implementations of DD-based analysis and specifying the problems they face. Recall that what I called DD-based approaches (i.e., analyses assuming that the protagonist’s perspective is rendered in FID by direct access to her context without any mediation through the narrator’s context) encompass two types of analyses: bicontextual analyses (Eckardt, 2014; Schlenker, 2004, i.a.), which we review in Sect. 3.1, and mixed quotation analyses (Maier, 2015, i.a.) discussed in Sect. 3.2.

### Bicontextual analyses

#### Schlenker (2004)

Building on Banfield (1982), Doron (1991) and Recanati (2000), Schlenker (2004) proposes that the grammatical notion of context of speech should be ramified into a context of thought and a context of utterance, and the specificity of FID (as well as historical present) is to distinguish between these two contexts: while the context of thought is anchored to the protagonist in FID, the context of utterance is anchored to the narrator. Sentences in FID must thus be evaluated with respect to two contexts. The division of labor between the two contexts, Schlenker argues, is determined by the distinction between truth and failure conditions: the context of thought contributes to the truth conditions while the context of utterance only contributes to the failure conditions. Specifically, the context of utterance only plays a role in the evaluation of the presuppositions associated with sorted variables—a class of elements that Schlenker argues (based on independent facts) is formed by tenses and pronouns.

Schlenker’s hypothesis thus derives and predicts (broadly correctly, as we discuss below) that tenses and pronouns are relativized to the narrator’s utterance context, while the rest (including adverbial indexicals) is relativized to the protagonist’s thought context. First, Schlenker (2004, pp. 286–288) shows independently of FID that both tenses and pronouns behave like sorted variables, because they can be bound or free, they can be responsible for referential failure and they can be semantically uninterpreted in focus alternatives; the first and the third properties are illustrated in (143).

**Table:** 

(143)	a.	Only Mary did her homework. [Therefore Peter didn’t do his] (Schlenker, 2004, pp. 286–288)
	b.	Only I did my homework. [Therefore Peter didn’t do his)
	c.	Only then did I work in Boston. [Therefore now I don’t work in Boston]

Assuming that the context of utterance υ (vs. the context of thought θ) only contributes to failure conditions, this gives rise to the following lexical entries for, e.g., the feminine third person pronoun and the past tense (with g the assignment function).

**Table:** 

(144)	a.	*she*_*k*_ denotes_g,υ,θ_ # iff g(x_k_) isn’t (in the world of υ) a female who is neither the speaker nor an addressee of υ. Otherwise it denotes_g,υ,θ_ g(x_k_).
		(Schlenker, 2004, p. 292)
	b.	*past*_*k*_ denotes_g,υ,θ_ # iff g(t_k_) isn’t before the time of υ. Otherwise, it denotes_g,υ,θ_ g(t_k_).

In other words, pronouns and tenses are anchored to the narrator because they are presuppositional and the narrator’s context is responsible for presuppositions (and only for them).

Second, all non-presuppositional elements are evaluated with respect to the context of thought. For example, adverbial indexicals are interpreted as in (145).

**Table:** 

(145)	a.	*here* denotes_g,υ,θ_ the location of θ.	
	b.	*tomorrow* denotes_g,υ,θ_ the day that follows the time of θ.	(Schlenker, 2004, p. 292)

The strength of Schlenker’s analysis is thus to derive (vs. stipulate) the perspectival differences observed in FID (esp. pronouns/tenses vs. adverbial indexicals) from an independent semantic difference (presuppositional variables vs. non-presuppositional elements) and to motivate the relativization to two contexts; in this respect, not only does the distinction between the context of thought and the context of utterance reflect the intuition that in FID, the protagonist’s thoughts are articulated through the narrator’s mouth, but Schlenker argues that the reverse image of FID (where it is the context of thought that is anchored to the narrator) is also attested with the historical present.

But this account faces three main types of problems. The first type of problem comes from the prediction that presuppositions are evaluated in the narrator’s context. As acknowledged by Schlenker, this raises some issues regarding the interpretation of some pronouns. In particular, we saw in Sect. 1.2.1 that the gender and number of at least some pronouns must be evaluated by the protagonist in FID (see, e.g., (16) and (18), where the protagonist is mistaken about the gender or number of some individual), while Schlenker’s account predicts gender and number features, just like person features, to be relativized to the narrator’s context. To remedy this problem, Schlenker (2004, p. 291) tentatively suggests that in those cases, pronouns can be read as hidden descriptions (or so-called e-type pronouns) such as *the man* or *the woman* as is independently attested (see Karttunen 1969, i.a.). But in the absence of a motivation for the parallel between two types of cases, this hypothesis seems ad hoc, as mentioned by Maier (2015, p. 356).

A similar solution is proposed by Schlenker (2004, p. 294) to tackle another problem regarding the rendering of *de se* thoughts in FID. Schlenker shows that the procedure he proposes entails that the sentence in (146)a (cf. (42)-(43)) expresses the thought in (146)b.

**Table:** 

(146)	a.	His_k_ pants were_m_ on fire (, John_k_ thought).
	b	T(a) = *λθ[has-pants-on-fire(x*_*k*_*, t*_*m*_*, actually) is true*_*s,θ*_*]*= *λθ*[g(x_k_)’s pants are on fire at time g(t_m_) in the world of θ].

(146)b is the set of thought contexts that make (146)a true; note that the context of utterance has been eliminated here by stripping the sentence of its person and tense features (assuming that no referential failure occurs). Crucially, the result fails to yield the desired difference between a *de se* thought (John thought *My pants are on fire*) and a non *de se* thought (John thought *His pants are on fire*). To solve the problem, Schlenker proposes to adapt Kaplan’s (1969) solution to Quine’s (1956) Ortcutt problem (i.e., Ralph believes of Ortcutt both that he—qua the man he saw in a brown hat—is a spy, and that he—qua the man he saw at the beach—is not a spy). Specifically, he suggests that the variable *x*_*k*_ can be replaced with a description under which the protagonist (i.e., the author of θ) is acquainted with the referent of *x*_*k*_—including the indexical description *I*, as shown in (147), which thus yields the *de se* reading of (146)a.

**Table:** 

(147)	T_1_((146)a) = *λθ’[has-pants-on-fire(I, t*_*m*_*, actually) is true*_*s,θ’*_*]*= *λθ’*[the pants of the author of *θ’* are on fire at time s(t_m_) in the world of θ].

But especially given that under Schlenker’s analysis, FID does not include any attitude operator (unlike in Ortcutt’s cases), this solution also seems ad hoc (see discussion in Sharvit, 2008, p. 372).

Furthermore, the same problem arises when the protagonist is referred to by a first or second person pronoun as in (19) or (27). This makes the problem even more acute. It implies that a first person pronoun (a variable associated with the presupposition that the referent is the speaker of υ) is understood as the description *the speaker of θ* (essentially promoting the presupposition to the assertion level and unshifting the indexical). But first and second person pronouns usually resist being interpreted as hidden descriptions except under some very specific circumstances (see Jacobson, 2012; Charnavel, 2019b, i.a.).

Further note that *de te* readings raise a similar problem. Recall that a third person pronoun (see fn. 6), a first person pronoun (see (20)) or a second person pronoun (see one possible interpretation of (28)) can refer to the addressee of the protagonist. But Schlenker’s procedure fails to distinguish between a *de te* reading (where the protagonist actually said *you*) and a non *de te* reading (where the protagonist did not recognize the referent as her addressee and thus referred to her by *she*; see fn. 12). The solution suggested by Schlenker for *de se* readings can apply here (e.g., *me* could be interpreted as *the addressee of θ*) but raises the same issues.

Moreover, the explanatory power of Schlenker’s analysis is weakened by the fact that not all presuppositions are evaluated uniformly with respect to the narrator’s context. As we saw, the main strength of this hypothesis consists in deriving the difference in behavior between tenses/pronouns and other elements from the independent difference between presupposition and assertion. But as observed by Abrusán (2021, p. 870), other presuppositions than those associated with pronouns are evaluated with respect to the protagonist’s context (cf. Eckardt, 2014, p. 61) as illustrated in (148).

**Table:** 

(148)	Tom frowned. *The ghost in the attic was making noises again*. (Eckardt, 2014, p. 60)

Here, only Tom needs to presuppose that there is a ghost in the attic that used to make noises, the narrator need not. This observation weakens the explanatory power of Schlenker’s analysis as the distinctive behavior of tenses and pronouns cannot be attributed to the distinctive behavior of presuppositions in general, but only to those associated with pronouns. This may be independently motivated, since as noted by Schlenker (2004, fn. 6), pronoun presuppositions (or referential failure for pronouns, in his terms) exhibit some specific behaviors as compared to other presuppositions, but this specificity remains poorly understood.

The second type of problem comes from the prediction that non-presuppositions are evaluated in the protagonist’s context. As shown by Maier (2015), this prediction is challenged by the fact that some proper names can appear in FID to represent a first or second person pronoun in the original speech or thought (cf. Fludernik, 1993, pp. 136–137, Authier-Revuz, 2020, p. 137), as illustrated in (149).

**Table:** 

(149)	He [=Arnie] dialed Leigh’s number from memory. Mrs Cabot picked the phone up and recognized his voice immediately. Her pleasant and rather sexy come-hither-thou-fascinating-stranger phone voice became instantly hard*. Arnie had had his last chance with her*, that voice said*, and he had blown it*. [King, *Christine*] (Maier, 2015, p. 358)

Here, *Arnie* in Mrs. Cabot’s FID stands for *you* in the DD counterpart (*you have had your last chance with her*). Maier suggests that the proper name is here used in place of a third person pronoun because the referent is not salient enough; more generally, proper names are used to avoid ambiguity. Such instances are unexpected under Schlenker’s account, where *Arnie* should be evaluated in the protagonist’s context, i.e., Mrs. Cabot’s, and thus correspond to her own words (while she in fact said *you*).^45^

A similar problem is created by the fact that, as we observed in Sect. 1.3.2, anaphoric adverbials are acceptable in FID (see, e.g., *le lendemain* ‘the next day’ in (100)), which cannot correspond to the exact words of the protagonist either (*Voyons, ce sera pour {#le lendemain/demain} dix heures* ‘Come on! it will be at ten o'clock {#the next day/tomorrow}’).^46^

More generally, the hypothesis that the protagonist’s words (except for tenses and pronouns) are recovered in FID (see Schlenker, 2004, pp. 295–296) is challenged by the observation that as described in Sect. 1.3.2, FID need not be a verbatim report, but can involve typicality (see (82)), word reformulation (see (84)) or style change (see, e.g., (86)).

The third type of problem is due to the assumption that FID does not involve any modal operator (Schlenker, 2004, p. 293); relativization to the protagonist’s thoughts is not achieved through scoping under an attitude operator, but through bicontextual dependency. This hypothesis creates difficulties for FID phenomena relying on attitude embedding described in Sects. 1.2.2 and 1.2.3. First, we observed that FID can be recursively embedded. But as acknowledged by Eckardt (2014, pp. 250–251), bicontextual accounts do not (at least straightforwardly) allow for recursive embedding.

**Table:** 

(150)	*Letting herself be helped by him*, Mrs. Ramsay had thought (Lili supposed) …* obviously it had*, Lily thought….

For example, Schlenker’s account predicts (67), partially repeated as (150), to be evaluated with respect to a context of thought and a context of utterance. We may assume that the context of thought is first anchored to Mrs. Ramsay, then to Lili, and the context of utterance is first anchored to Lili and then to the narrator. But first, it is not obvious how the system can achieve such anchoring shift (in the absence of explicit explanation about the identification procedure for context authors). Second, even granting this, the first FID sentence would not express any thought embedding (and relativization to more than two contexts would considerably complexify and undermine the whole system). Worse, it would wrongly predict that a first person pronoun could refer to another individual than the narrator in FID (i.e., Lili here) (cf. Eckardt, 2014, p. 58).

Second, we observed that FID licenses logophoric elements, and excludes antilogophoric elements. It is unclear how Schlenker’s system could allow for the use of logophoric *himself* in (50) (repeated below).

**Table:** 

(151)	*That was one of the bonds between Sally and himself*.

Schlenker’s account is incompatible with any analysis requiring a logophoric operator here, as FID is assumed not to involve any modal operator; in fact, Schlenker (2005, p. 62) explicitly states that his account predicts that logophors cannot refer to the FID protagonist. It could perhaps exploit the hypothesis that only *him* is evaluated in the narrator’s context, while *self* is evaluated in the protagonist’s context (assuming a DD counterpart involving *myself*). But this would ignore the logophoricity of *himself*, thus leaving its general distribution unexplained.

The unacceptability of antilogophoric elements in FID challenges Schlenker’s account even more clearly. Recall that epithets referring to the protagonist (as in (62) repeated in (152)) and antilogophoric pronouns like French *en* (as in (64) repeated in (153)) are incompatible with FID.

**Table:** 

(152)	Lucy_i_ was very worried. [The poor woman]_*i/k_*’s parents would not listen to her*.
(153)	Emile_i_ exultait. *Oui, c’était sûr maintenant, Sophie*_*m*_ en_*i/k_ *était amoureux!*
	‘Emile_i_ was exulting. *Yes, it was now certain, Sophie*_*m*_* was in love with him*_*i/k*_*!*’

Under Schlenker’s hypothesis, *the poor woman* in (152) is predicted to be evaluated by the protagonist and thus presumably expected to be acceptable if Lucy judges herself as a poor woman, contrary to fact. Unacceptability may be attributed to the infelicity of a DD counterpart using a bare epithet ([The poor woman]_*i/k_*’s parents won’t listen to me*); but this would require assuming that FID must be a verbatim report, which we saw above is problematic. Even more clearly, *en* in (153) is incorrectly predicted to be acceptable under Schlenker’s account as pronouns are evaluated in the narrator’s context; coding antilogophoricity as a presupposition would not work as pronoun presuppositions should be relativized to the narrator’s context, while antilogophoricity would require making reference to the protagonist’s context. Note that for the same reasons, reflexives bound *de re* as in (54) and *come* anchored to the narrator’s context as in (59) are incorrectly predicted to be acceptable under bicontextual accounts.

#### Eckardt (2014)

Eckardt’s (2014) goal can be seen as broadening the empirical coverage of Schlenker’s analysis. She basically adopts his bicontextual analysis, but modifies it so as to make correct predictions about phenomena that are not examined in Schlenker (2004), such as aspect, discourse particles and exclamatives. Specifically, Eckardt proposes that sentences can be interpreted in two modes: the normal (i.e., non-FID) mode relativized to only the external context of evaluation C (〚S〛^M, g, C^), and the FID mode relativized to two contexts of evaluation, C and the internal context c centered on a protagonist (〚S〛^M, g, <C,c>^). The difference between indexicals that do not shift in FID (e.g., first and second person pronouns) and those that shift (e.g., adverbial indexicals) is coded in the lexical entries of these items. For instance, the singular first person pronoun is interpreted as in (154), while *here* is interpreted as in (155).

**Table:** 

(154)	a.	〚*I*〛^M, g, C^ = 〚sp〛^M, g, C^ = C(sp)
	b.	〚*I*〛^M, g,<C,c>^ = 〚sp〛^M, g,<C,c>^ = C(sp)
(155)	a.	〚*here*〛^M, g, C^ = 〚here〛^M, g, C^ = C(here)
	b.	〚*here*〛^M, g,<C,c>^ = 〚here〛^M, g,<C,c>^ = c(here)

Thus, non-shiftable indexicals like *I*, noted in small capitals, are always evaluated with respect to C, while shiftable indexicals like *here*, noted in lower case, are interpreted with respect to C if there is only one context, but with respect to c in bicontextual mode.

The lexical coding of shiftability allows Eckardt to accommodate the behavior of discourse particles within her system. Discourse particles are usually assumed not to contribute to the assertive level, but to the presuppositional level or the expressive content of an utterance (see review in Zimmermann, 2011). Nevertheless, Eckardt (2014) shows that they are anchored to the protagonist in FID, contrary to what would be expected under Schlenker’s system. Her system can lexically encode shiftability in any semantic level of the lexical entries.

Furthermore, Eckardt (2014, Ch. 4) introduces reference times in order to capture the distribution of aspect in FID. Reference time (cf. Reichenbach, 1947) corresponds to the time of interest, which is distinct from the event time—as reflected by aspect—and distinct from the utterance time—as reflected by tense. Their incorporation into the system allows Eckardt to explain the constraints on combinations between temporal adverbials and tense/aspect. Like Schlenker, she hypothesizes that adverbial indexicals depend on the protagonist’s time (*now*), while tense relies on the narrator’s time (*now*) (cf. (144)b-(145)b). But Eckardt also relativizes tense to the reference time (and adopts Davidson’s, 1967 use of events) and defines aspects with respect to a time of interest. She can thus derive the FID interpretation of sentences like (156) (see fn. 34) adopting Doron’s (1991) generalization that the narrator’s reference time R in FID coincides with the time of the protagonist (C(r)=c(now)).

**Table:** 

(156)	*Antje had bought a dress yesterday*. (Eckardt, 2014, p. 97)

The use of *yesterday* here implies that the event of Antje buying a dress takes place one day before the protagonist’s time of utterance. Perfective aspect further indicates that it takes place before the time of interest, which corresponds to the protagonist’s time of utterance due to Doron’s generalization. As for past tense, it implies that this time of interest is before the narrator’s time of utterance. All this is coherent and more fine-grained than Schlenker’s predictions that Antje’s time of buying a dress occurs before the narrator’s time of utterance and one day before the protagonist’s (cf. Eckardt, 2014, pp. 185–188).

Furthermore, Eckardt proposes that the reference time is context-based and can be relativized to the protagonist in some cases such as exclamatives or German *Konjunktiv*. Inspired by Rett (2008), she assumes that exclamatives express temporally anchored degrees; in FID, exclamatives must thus refer to the shifted reference time r (as the degree can only be witnessed as long as the event takes place). Similarly, Eckardt (2014, Ch. 8) assumes that German *Konjunktiv* tenses are relativized to the shifted reference time r (vs. other tenses). Schlenker’s analysis, however, seems incompatible with the use of *Konjunktiv* in FID, since tenses must be evaluated with respect to the narrator in Schlenker’s system, while the use of *Konjunktiv* is restricted to reported contexts (see Sect. 1.2.2). For Schlenker (2004), it thus raises the same problem in the domain of moods as logophoric pronominals in the domain of person (see Sect. 3.1.1).^47^

Finally, Eckardt shows that Schlenker (2004) incorrectly predicts the unacceptability of FID in historical present. According to Schlenker (2004), the historical present is the mirror image of FID, in the sense that the context of thought corresponds to the narrator’s actual context (thus attributing the assertion to the narrator) and the context of utterance to some other (past) context (thus licensing present tense and yielding the impression that the speaker directly witnesses the events reported). For example, in (157), the context of utterance is set 58 years before the narrator’s context of thought, which explains the co-occurrence of the present tense and the adverbial *fifty-eight years ago*.

**Table:** 

(157)	Fifty-eight years ago to this day, on January 22, 1944, just as the Americans are about to invade Europe, the Germans attack Vercors.

But Eckardt observes that this account cannot derive that FID can occur in stories told in the historical present:

**Table:** 

(158)	Alice is looking at the White Rabbit with amazement. *How tall it is!*

Under Schlenker’s (2004) account, the use of the historical present in (158) contradictorily implies that the context of thought is the narrator’s, while FID implies it is the protagonist’s.^48^

In sum, Eckardt (2014) basically adopts Schlenker’s (2004) bicontextual approach, but broadens its empirical coverage and overcomes some of its problems by enriching it. Nevertheless, Eckardt’s approach remains problematic: not only does it not solve all of Schlenker’s problems, it also loses its explanatory power. We saw that one of the strengths of Schlenker’s analysis is deriving the perspectival division of labor between pronouns/tense and other elements from an independent difference between presupposition and assertion. To correct the empirical problems (reviewed above) this proposal raises, Eckardt instead codes contextual anchoring in lexical entries. We have already discussed cases involving person and location or time indexicals, discourse particles, tenses and moods. Similarly, Eckardt proposes capturing cases of mistaken gender like (16) using the following lexical entry, which relativizes gender belief to the protagonist (*sp*), and identity belief to the narrator (*sp*).

**Table:** 

(159)	〚*she*_*j*_〛^M, g, <C,c>^ = x_j_	
	presupposition 1: Belief (*sp, w, λw’*.Female*(x)*)	
	presupposition 2: *x *≠ *sp*	(Eckardt, 2014, p. 195)

But the behavior of pronouns is thereby simply stipulated (and it does not capture the difference observed in (16) with respect to gender between pronouns referring to the protagonist and pronouns referring to other individuals). The same kind of stipulation is required to capture the distribution of imperatives or vocatives. In general, Eckardt’s account does not thus derive the constraints on perspectival anchoring in FID, but lexically states them. If it certainly improves on Schlenker’s hypothesis with respect to empirical adequacy, it thereby loses predictive power.

Furthermore, Eckardt (2014) does not correct all wrong predictions of Schlenker’s account. First, Eckardt (2014, pp. 57–59, 250–251) acknowledges that her approach does not allow for recursive embedding as in (150). Eckardt (2014, Ch. 3) hypothesizes that the belief ascription of FID sentences to the relevant protagonist (instead of the narrator) happens at the level of story update, which is based on Stalnaker’s (2002) pragmatic rules. It may thus be assumed that in relevant circumstances such as (150), these rules ensure that the anchoring of the internal context c switches from Lili to Mrs. Ramsay (say, from <C, c> to <C, d>). But there is no formal way in this system—or in any bicontextual account—to encode thought (or speech) embedding (*Lili thought that Mrs. Ramsay thought that…*).

Second, the acceptability of logophoric elements and the ban on antilogophoric elements when anchored to the protagonist (see (151)-(153)) is problematic for Eckardt’s account. As mentioned above, it is possible for her to lexically encode dependency on the protagonist. But this proposal is doubly stipulative. First, as already mentioned, it simply states (instead of explaining) that some elements (e.g., *Konjunktiv*) are anchored to the protagonist, while others (e.g., indicative) are anchored to the narrator. Second, this account, which relies on double context dependency, can only be applied to FID. But as mentioned in Sect. 1.2.2, logophors are characteristic of embedded contexts, including both FID and ID. Thus, *Konjunktiv I* also appears in ID (as in, e.g., (65); see Fabricius-Hansen & Sæbø 2004). But under Eckardt’s account, only FID relies on bicontextual evaluation, so that it cannot extend to *Konjunktiv I* in embedded clauses (as acknowledged by Eckardt, 2014, p. 213, fn. 5). The same problem arises for all logophoric elements: Eckardt’s approach implies that their occurrence in ID and in FID cannot receive the same explanation contrary to theoretical economy.

Third, we have observed that in FID occurring in communicative contexts like (101), adverbial indexicals can (or even must, depending on cases) be anchored to the narrator. As acknowledged by Eckardt (2014, p. 217), this fact cannot be predicted by her account, which implies that all shiftable indexicals must shift when evaluated in the bicontextual mode of interpretation.

In sum, bicontextual accounts face important empirical and theoretical problems. The main empirical problems concern the explanation of recursive embedding and the distribution of (anti)logophoric elements in FID. The main theoretical problem consists in the stipulations that are required to correctly capture the perspectival division of labor between various elements in FID.

### Mixed quotation analyses

An alternative to bicontextual accounts is proposed by Maier (2015, 2017) (cf. Deal, 2020, pp. 111–115) in terms of mixed quotation. Mixed (vs. pure) quotation refers to quotation in which a phrase is simultaneously used and mentioned, as in typical DD. Under Maier’s approach, FID is thus an instance of DD in which some elements (typically pronouns and tenses) are unquoted as schematized in (160) (using editorial typographic conventions according to which brackets mark unquotation and quotation marks indicate DD).

**Table:** 

(160)	“Tomorrow [was] Christmas!”

Ignoring details of formalization here (which are irrelevant to our purposes), the protagonist’s thought represented by FID is captured as DD where the verb is unquoted (and thus in past tense). More generally, FID is conceived as DD with holes for tenses and pronouns, which are unquoted. According to Maier (2015), this hypothesis is motivated by the fact that unquotation is independently attested overtly in certain genres of factual reporting.

As acknowledged by Maier (2015, pp. 367–368) and mentioned by Abrusán (2023, p. 134), this extension of (un)quotation to FID requires two nontrivial adjustments. First, (un)quotation must be able to cover mental acts that are not uttered, while (un)quotation is otherwise typically attested in cases of speech reports. This adjustment is all the less trivial as it seems to correlate with a difference in communicative purposes and effects between journalistic (un)quotation and FID (whose precise examination would lead us too far afield, but see some ideas in Eckardt, 2014, pp. 197–198). Second, it must be assumed that the logical form includes a hidden attitude operator to correctly attribute the mental act to the protagonist (vs. the speaker). This second move is also necessary to correctly capture the distribution of tenses and *de se* (and *de te*) readings (see Maier, 2015, fn. 39), as well as, presumably, logophoric elements (which are not discussed by Maier).^49^ This aspect of the analysis likens it to ID-based analyses, thus weakening its competitiveness as a DD-based analysis.

Even granting these points, the mixed quotation approach, like the bicontextual accounts it aims at improving upon, faces both theoretical and empirical challenges. The theoretical challenge is one of predictive power. Maier (2015) assumes that tenses and pronouns are by and large anchored to the narrator due to unquotation. Similarly, this account, unlike Schlenker’s, is compatible with the occurrence of proper names referring to the protagonist in FID as in (149), as long as it is assumed that proper names can also be unquoted. The same could presumably hold of anaphoric adverbials as in (99)-(100), which also challenge Schlenker’s proposal. But in the absence of any independently motivated principle regulating the use of unquotation, this solution is stipulative and lacks predictive power: any expression could be quoted or unquoted, predicting any possible combination, while FID is in fact constrained with respect to the perspectival division of labor between the narrator and the protagonist.

Aware of this problem, Maier (2017) strives to formulate such an independent pragmatic principle responsible for unquotation, based on cases of apparent unquotation in direct discourse constructions (including data from Kwaza speakers, Catalan signers, and Dutch children). Namely, unquotation arises as a solution to the conflict between two contradictory constraints: a bias for anchoring indexicals to the most salient speech act (attraction), and a bias for faithfully reproducing the linguistic form of the reported utterance (verbatim). Conflicts specifically arise when the quoted speech act involves someone who plays a different role in the actual speech act; unquotation resolves the conflict by locally suspending the verbatim constraint. This proposal directly applies to FID examples where in the DD counterpart, the FID first person pronoun corresponds to a second or third person pronoun (see, e.g., (20)-(22)), or the FID second person pronoun corresponds to a first or third person pronoun (see, e.g., (2), (27)). But in many cases of FID, the unquoted pronouns refer to the story’s protagonist, which is both physically and temporally removed from the actual speaker, i.e., the narrator. In those cases, Maier argues that the same principle is at play, that is, attraction overrules verbatim, but the difference is that the source of attraction is the protagonist and her here and now instead of the actual speech act. This results from the fact, so the argument goes, that in stories told by an omniscient narrator, the protagonist is more salient than the narrator, and the story time is more salient than the time of the narration. This is why pronouns and tenses are generally unquoted in FID.

But Maier’s (2017) proposal faces several challenges. First, cases of first or second person pronouns co-occurring with past tense (as in (20)-(22) or (27)) do not straightforwardly follow, as the form of the pronouns implies that the source of attraction is the actual speech act (when the protagonist and the narrator are distinct), while the past tense implies that it is the story’s time. Second, Maier (2015) argues that partial unquotation can happen in cases where the FID *he*, corresponding to DD *you*, reflects the protagonist’s judgment about gender, but the narrator’s about person (see fn. 6): gender features are quoted while person features are unquoted. It’s unclear how such partial unquotation can result from Maier’s (2017) pragmatic principle, since the resulting *he* respects neither verbatim nor attraction. And even granting that this may somehow be a solution of compromise, it begs the question of the modalities of application of the principle: under what conditions does attraction win? When does verbatim win? When does a compromise solution obtain? These questions are especially pressing in the time domain. To account for the use of past tense, it is assumed that attraction overrules verbatim, and to account for the use of time indexicals anchored to the protagonist, it is assumed that verbatim wins. Why does the pragmatic principle apply differently to tense and to indexicals? Furthermore, the case of anaphoric adverbials (e.g., *the next day*) as in (99)-(100) should be assumed to result from attraction. The flexibility required for this pragmatic principle to correctly capture some facts undermines its predictive power. Worse, it makes incorrect predictions. It is thus never the case that the protagonist can be referred to as a first person pronoun in FID if it is distinct from the narrator. But this is predicted to be possible as long as verbatim overrules attraction as happens with location and time indexicals.

Further incorrect empirical predictions, outside the domain of tense and pronouns, are made by Maier’s account, which further question the possibility of independently motivating appropriate conditions of application for the pragmatic principle of unquotation. First, the ban on vocatives and imperatives (or at least their restricted distribution, see Sect. 1.2.1) remains unexplained under a quotation account. Why couldn’t vocatives and imperatives be quoted? If attraction is to blame, how exactly can it work so as to not lead to an overapplication of attraction against verbatim? Second, the possible stylistic manipulations of the narrator observed in 1.2.4 raise the reverse problem. In fact, Maier (2015) presents the possible occurrence of the protagonist’s dialect in FID as an argument for the quotation account. Why can some formulations be unquoted, on the contrary? In what sense can attraction apply in such cases? This is especially puzzling given the observation that *de re* non *de dicto* expressions are uncontroversially precluded in FID, as we saw in Sect. 1.1.2. The question is even more acute when the reformulation does not target only one word or expression as in (84), but whole sentences as in (19) or (86), which are intended to reflect the protagonist’s (e.g., the child Proust) thought, but not his manner of speaking. In those cases, neither quotation (which should entail a faithful rendering of the style), nor unquotation (which should entail belief attribution to the narrator) can correctly predict the facts. In sum, not only is it difficult, under quotation accounts, to explain (vs. stipulate) the division of labor between quotation and unquotation in FID, it is also impossible to account for cases where some aspects of both are required.

Further notice that the same kinds of problem arise if we try to motivate a principle for quotation instead of unquotation, as Eckardt (2014, pp. 197–204) argues. In particular, she observes that in ordinary quotation, words and their features cannot be split up so that the morphophonological form of words is quoted while the features (or even only some of the features) are unquoted, as we saw should happen in FID for tense and pronouns. Moreover, it is virtually impossible in ordinary quotation to quote isolated particles, and fully impossible to quote tacit operators such as question or exclamative operators as should be allowed in FID (e.g., German *Sie liebted ihn ja* ‘she loved him *ja*’ or English *Was she?*).

Finally note that recursive FID embedding is problematic under quotation accounts, though perhaps not fully impossible. Certainly, DD within DD is possible, and the silent attitude operators posited by Maier can in principle be stacked. But while quotation seems to be recursively embeddable, unquotation does not, at least not in the appropriate way (e.g., Mary said: “John begged me: ‘{you /[I]/*[she]} should come tomorrow’”).

## Conclusion

This article has made a case for a logophoric analysis of FID. The hypothesis that FID passages are under the scope of a logophoric operator straightforwardly accounts for several properties that remain unexplained under bicontextual or quotation analyses: most strikingly, the licensing of logophoric elements and the ban on antilogophoric elements in FID, as well as the acceptability of recursive FID. Instead of being conceived as an idiosyncrasy, FID is thereby classified under an independently motivated linguistic category, and can be treated uniformly with other similar phenomena such as logophoric contexts or Protagonist Projection. Literary effects derive from the exploitation of this linguistic mechanism licensing the expression of various perspectives in a constrained way.

## Footnotes

1 It seems that the category of FID was created by the Swiss: Adolf Tobler gave the first definition of FID in 1887 and Charles Bally coined the French term of *style indirect libre* in 1912 (see Philippe & Zufferey 2018). Note that here, I cannot do justice to the rich stylistic literature on FID in various linguistic traditions and literary criticism (see, e.g., Cohn 1978; Fludernik 1993 and references therein).

2 For clarity, I will henceforth italicize all parts of examples that I interpret as FID. Further note that I indicate literary sources in small font between brackets, and linguistic sources in normal font in parentheses. English translations (from French, German, Italian or Hebrew) are either the linguistic author’s or mine.

3 Other languages than the language of the narration are usually assumed not to appear in FID (see Authier-Revuz 2020, i.a.). Some examples can nevertheless be found as in *Under the Volcano* by Malcolm Lowry, where some FID sentences are in Spanish while the rest of the narration is in English.

4 The last point is questioned by Schlenker (2004); although he agrees with the judgment in (16)b, he reports the following judgment for (i):

**Table:**

(i)	[In John_i_’s dream, Ann_k_ was an old priest that he had known for ages.]
	*Really*, John_i_ thought in his dream, {*?**she*_*k*_*/#**he*_*k*_}* was now a terrible priest.*

5 Cases where the relevant pronoun refers to the protagonist’s addressee are more controversial: while Maier (2015, p. 370) suggests that its gender must reflect the protagonist’s judgment, Cumming and Sharvit (2016, fn. 11) claim that it must refer to the referent’s true gender. The log-FID analysis (see Sect. 2.1) predicts that in that case and gender features must reflect the narrator’s judgment if the pronoun is read *de te* (cf. Cumming & Sharvit 2016), and the protagonist’s judgment otherwise (see fn. 12), which is consistent with my own judgments.

6 It is difficult to judge cases of mistaken number for pronouns intended to refer to the protagonist, as in (ii):

**Table:**

(ii)	Fred was so drunk that when he looked at himself in the mirror, he perceived himself as two guys. *How strong {?they were/?he was}! {?They/?he} would definitely win tomorrow* This difficulty may come from the *de se* requirement (see (42)): if Fred perceives himself as two men, he can’t think of himself as *I*, but as *we*, which thus includes both a *de se* component and a non *de se* component (Fred thinks of himself as *I + him*).

7 According to Sharvit (2008, p. 358, fn. 3), at least some informants accept a first person pronoun in FID, even if the speaker is not an addressee of the FID protagonist. But she does not detail under what conditions.

8 Note that the use of French impersonal *on* in FID raises further questions. Outside FID, *on* exhibits a (colloquial) use as a first person plural pronoun (besides existential and generic—speaker-inclusive or—exclusive—uses; see, e.g., Creissels 2008), as shown by the possible coreference with an actual first person plural possessive *nos* in (iii).

**Table:**

(iii)	On verra bien demain! On pourra reparler de nos problèmes à ce moment-là.
	‘We’ll see tomorrow! We’ll talk again about our problems then.’ Interestingly, the first person plural pronoun use seems to be attested in FID (cf. Fludernik 1993, pp. 117-118), as shown in (ii), where count Muffat discusses a difficult matter with Nana, who is sick.

**Table:**

(iv)	Il la calma, en lui prenant une main. *Oui, l’**on** verrait, l’important était qu’elle se reposât*. [Zola, *Nana*]
	‘He took her hand and soothed her. *Yes, **he** would see about it; the important thing now was for her to rest.*
	[translation from the Project Gutenberg www.gutenberg.org] As indicated by the English translation *he* (an alternative could have been *they*), *on* is here intended to include the protagonist (Muffat) in its reference, but not the narrator. At first glance, this seems to go against the generalization that person features are relativized to the narrator in FID; in other words, *on* seems to shift to the protagonist here. However, we also observe that a transposition of (iii) to FID is inexpressible: *on* is acceptable in FID, but the co-occurrence with *nos* is not transposable in FID.

**Table:**

(v)	Elle s’impatientait devant l’insistance de son interlocuteur. *On verrait bien demain! On pourrait reparler de {*nos/*leurs} problèmes à ce moment-là.*
	‘She grew impatient with her interlocutor’s insistence. *They would see about it tomorrow! They could talk about their problems then.*’ This suggests that the interpretation of *on* as a first person plural pronoun does not necessarily require positing first person features for *on*.

9 Banfield (1982) incorrectly assumes that present tense is prohibited from FID.

10 Although Eckardt (2014) does not discuss embedded uses of discourse particles in ID, she mentions an example with embedded *wohl* (Eckardt 2014, p. 129). See, e.g., Zimmermann (2011) for a review of discourse particles, including a discussion on their embeddability.

11 The same holds of *de te* readings. Pronouns in ID can be read *de te* or not (see vii), while it seems difficult to read a pronoun non *de te* in FID if the narrator specifies the identity mistake as in (vi)a vs. (vi)b.

**Table:**

(vi)	a.	At an association meeting, a group was talking about [the president of the association]_i_. Mary, a new member of the association, turned to a man next to her, who was in fact the president, unbeknownst to her. #*Where was he*_*i*_*? She had several complaints to make,* she asked.
	b.	At an association meeting, a group was talking about [the president of the association]_i_. Mary, a new member of the association, turned to [a man next to her]_k_. *Where was he*_*i*_*? She had several complaints to make,* she asked. In fact, her addressee was the president himself.
(vii)		Mary asked the President where he was.

12 As noted in e.g., Banfield (1982, p. 104), the French *passé simple* (historic preterite or aorist) seems to be precluded from FID (see also discussion in Bres 2018; Gollut & Zufferey 2021, i.a.); only the imperfective *imparfait* is used as past tense in French FID. Except for Doron (1991, p. 60; cf. Giorgi 2010, pp. 186, 200 for Italian perfective tenses), both DD-based and ID-based accounts leave this fact unexplained. Informally, this ban on *passé simple* is due to its aspectual specificity, which induces that the event happened before the reference time (while the *imparfait* encodes that the event happens within the reference time), which must itself overlap with the actual utterance time; this creates a conflict given that the reference time must correspond to the protagonist’s utterance time in FID (Doron 1991). We will suggest in Sect. 2.2 (see fn. 34) how a logophoric approach imposing the protagonist’s perspective in FID may derive this fact.

13 Hacquard (2011, i.a.) suggests that sequence of tense may also affect epistemic modals in FID; according to her, it is for that reason that matrix epistemic modals can be evaluated under tense only in FID.

14 Sharvit (2010) reports that the first person reflexive is however acceptable in this scenario: The judgement of this example is however difficult. See fn. 19 for further discussion.

15 A reviewer wonders whether the juxtaposition of *come* and *there* (vs. *here*) induces a clash in perspective. Logophoric contexts certainly require homogeneous perspectives, but in the sense that all logophoric elements must be anchored to the same logophoric center (e.g., (93); see Charnavel 2019a, i.a.). The facts about indexicals in FID that we will review in Sect. 1.3.2 show, however, that indexicals and logophoric elements need not be evaluated under the same perspective (because indexicals do not shift in logophoric contexts, as we will argue in Sect. 2.3): indexicals can be anchored to the context of the narrator or the protagonist (depending on the pragmatics of contexts that we will discuss in Sect. 2.3), while logophoric elements like *come* must be anchored to the protagonist. The logophoric hypothesis thus correctly predicts that both *come there* and *come here* are infelicitous not because of a clash in perspective, but because the protagonist is not in the relevant place (*come* presupposing that the logophoric center is at the goal of motion). The fact that *come here* is not felicitous either further shows the narrator cannot be interpreted as the logophoric center in FID (and that *come* is not an indexical but a logophoric element, see Charnavel 2018, 2019a, i.a.).

16 The same holds of the other French anti-attitudinal French pronouns *y*, *ce* and *le lui* mentioned above in the text:

**Table:**

(ix)	a.	Emile_i_ s’inquiétait. **Sophie y*_*i*_* penserait-elle quand il*_*i*_* serait parti?*
	b.	Emile_i_ s’inquiétait. *Sophie penserait-elle à lui*_*i*_* quand il*_*i*_* serait parti?*
		‘Emile_i_ was worried. *Would Sophie think about him*_*i*_* when he*_*i*_* would be gone*?
(x)		Emile_i_ exultait. *Oui, {#c*_*i*_*’/il*_*i*_*} était un génie*!
		‘Emile_i_ was exulting. *Yes, he*_*i*_* was a genius*!
(xi)	a.	[L’enfant]_i_ s’inquiétait. **Est-ce que les maîtresses allaient le*_*i*_* lui confier, à la nouvelle assistante?*
	b.	[L’enfant]_i_ s’inquiétait. *Est-ce que les maîtresses allaient le*_*i*_* confier à la nouvelle assistante?*
		‘[The child]_i_ was worried. *Would the teachers entrust him*_*i*_* to (her –) the new assistant*?

17 *Now* in *the time has come now* is also anchored to Mrs. Ramsay’s temporality, but the FID status of this segment is less clear because of the present tense, suggesting that this clause may be DD rather than FID (like *I will marry you* later on in the passage).

18 This hypothesis may provide an explanation for Sharvit’s (2010) reported contrast between *de re* first and third person reflexives bound by PRO in FID (see fn. 15). Speakers who accept first person *myself* may construe the speaker as an FID protagonist through recursive FID embedding.

**Table:**

(xii)	*Ah! To hear **myself** sing again!* (I had thought, I then remembered.) Note that the second person reflexive (not mentioned in Sharvit 2010) seems to be licensed similarly:

**Table:**

(xiii)	A month ago, when you woke up from a coma, you didn’t know who you were. You listened to an old recording of yourself, not recognizing your own voice. Suddenly, the radio stopped playing, and you were frustrated that you couldn’t make it play again. *Ah! To hear **yourself** sing again!* (I guessed you (had) thought/you then told me). In the case of third person reflexives, however, fiction allows the (potentially omniscient) narrator not to provide evidence for their claim, which makes inappropriate the construing of the narrator as logophoric FID center through recursive embedding.

19 Banfield (1982) claims that the manner of speaking of the protagonist can only appear in FID representing speech (vs. thought) and only sporadically. Authier-Revuz (2020) claims that FID cannot appear in a different language than the narration (see fn. 4).

20 Conversely, Currie (2010) suggests that the narrator’s style may transpire by caricatural imitation of the protagonist’s style, which may create irony effects. For example, Currie (2010, p. 143) argues that in (xiv), stylistic imitation partly serves to ironize the perspective of the witness by using their uneducated speech recollecting events in their own community with a conventional emphasis in the exposition of the horrors.

**Table:**

(xiv)	People still remembered last year at Belchamp St Paul *how a strange gentleman came one evening in August years back; and how the next morning but one he was found dead, and there was an inquest; and the jury that viewed the body fainted, seven of ’em did, and none of ’em would speak to what they see, and the verdict was visitation of God; and how the people ’as kept the ’ouse moved out that same week, and went away from that part.* But they do not, I think, know that any glimmer of light has ever been thrown, or could be thrown, on the mystery. [*Count Magnus* 1931, M. R. James] By simply displaying the words, DD, Currie argues, would not achieve the same effect.

21 Conversely, syntactic embedding does not entail attitude embedding. This is for instance the case under modals (e.g., *it is likely that*) or non-attitude verbs, such as French *mériter* ‘deserve’ (see, e.g., (63), (140)b), which take a complementizer without introducing another attitude.

22 In fact, Maier (2015, p. 368, fn. 15, 39) also has to postulate the presence of such an operator, even under his mixed quotation analysis (see Sect. 3.2).

23 Sharvit (2008), who is the only defender of an ID-based analysis, also endorses this description and therefore has to stipulate that the FID operator quantifies over contexts (and assignment functions) and that there are two types of indexicals: those that never shift (person indexicals) and those that can shift (adverbial indexicals). These assumptions undermine an ID-based analysis in most subsequent views (e.g., Eckardt 2014, pp. 193-194; see Sect. 2.3).

24 Gollut and Zufferey (2021, p. 75) interestingly note that indexical (vs. anaphoric) adverbials in FID are not found before the 19th century in French. For example, La Fontaine’s *Fables*, which clearly involve FID, do not exhibit adverbial indexicals anchored to the protagonist in FID (see further discussion in fn. 42).

25 Further note that (99)-(100) cannot be treated as cases of Protagonist Projection (see discussion in Sect. 2.4), where adverbial indexicals do not shift, unlike in FID (see Abrusán 2021, i.a.): Protagonist Projection expresses preverbal perception, while (99) seems to correspond to Charlotte’s conscious recollection, and (100) clearly reports speech.

26 Sharvit (2008, fn. 4) notes that her FID operator is borrowed from Schlenker (1999). Note though that Schlenker (1999, p. 39) explicitly rejects the idea of a concealed attitude operator to account for FID, but hypothesizes that FID involves an utterance whose matrix context does not correspond to that of the actual speech act; this aspect will be incorporated in our hypothesis (see fn. 39). Doron (1991) proposes relativizing meaning in FID not only to *discourse* (speaker’s context) but also to *point of view* (protagonist’s context). Doron’s *point of view*, to which demonstratives and temporal indexicals (vs. person indexicals) are sensitive, is also a precursor of the FID operator. Our log-FID analysis is similarly inspired by Doron’s *point of view*, but bicontextual dependency will be abandoned (see Sect. 2.3).

27 Abrusán (2023, p. 138) shows that this anaphora resolution is more complex in some cases involving plural protagonists. For instance, (xv) requires e-type anaphora (*they* is interpreted as all the kids in town that were excited about Christmas).

**Table:**

(xv)	Most kids in town were excited about Christmas. *Santa Claus was coming!* (they thought)

28 Given that the attitudinal operator we posit for FID only gives rise to semantic (vs. syntactic) embedding, the argument based on imperatives and vocatives nevertheless forces us to commit to an analysis of the incompatibility of vocatives and imperatives in ID that exploits the properties of semantic (vs. syntactic) embedding. Put it differently, the unacceptability of imperatives and vocatives in FID may provide an argument for semantic vs. syntactic analyses of their incompatibility with ID.

29 *De te* pronouns can be similarly derived if we assume a further argument slot denoting the addressee on the FID operator (and accordingly amend the semantics for the operator) as well as a fourth lambda-operator in the complementizer binding an addressee pronoun (cf. Sharvit 2008; Cumming & Sharvit 2016).

30 Sharvit (2008) relies on the hypothesis that her FID operator quantifies over context-assignment pairs to derive this property, by assuming that world variables are indexicals that must shift. As discussed below in the text, Sharvit’s quantification over contexts will not be adopted, because it does not correctly derive the behavior of indexicals. Instead, Charnavel’s (2019a, 2020) approach is adopted here, under which world variables are always locally bound by local logophoric operators. Note that under this account, non *de dicto de re* readings in attitude contexts derive from the insertion of a logophoric operator anchored to the speaker in the relevant DPs; in this view, FID passages are characterized by the fact that no logophoric operator anchored to the speaker can occur within them; FID is thus a category only descriptively, not analytically.

31 Conversely, the hypothesis makes a prediction for gender ascription in ID. While both gender judgments (by the attitude holder or by the speaker) are possible in ID in general (see (17)), only the attitude holder’s judgment should be available in an attitude context containing a logophoric element such as an exempt anaphor. This seems correct:

**Table:**

(xvi)	a.	Mary_i_ was getting angry at Robin_k_, who she_i_ wrongly believed to be a man. She_i_ thought that
		{she_k_/he_k_} had destroyed the new pictures of her_i_.
	b.	Mary_i_ was getting angry at Robin_k_, who she_i_ wrongly believed to be a man. She_i_ thought that {#she_k_/he_k_} had destroyed the new pictures of herself_i_.

32 As discussed below, Sharvit’s hypothesis crucially differs from ours in that her FID operator also quantifies over contexts (and cannot thus be assimilated to a logophoric operator). In her system, the variable assignment can thus be relativized to the (internal) context. As log-FID does not quantify over contexts, we instead relativize the assignment to the attitude holder’s beliefs. And to obtain obligatory relativization to the attitude holder’s beliefs in FID (vs. ID), we must assume a mechanism similar to the one used for obligatory *de dicto* reading (see (115)), e.g., obligatory binding of assignments functions under log-FID.

33 The log-FID hypothesis may also provide a way to derive (vs. stipulate) Doron’s generalization that in FID, the reference time must correspond to the protagonist’s utterance/thought time, which accounts for aspectual restrictions. As observed by Doron and Eckardt, the perfective aspect in (xvii) (see (156) in Sect. 3.1.2) implies that the event took place before the protagonist’s thought time. Assuming Sharvit’s treatment of tense, the past tense comes from feature deletion under binding: the log-FID operator takes a time argument that binds the tense variable. We may further assume that it also binds the reference time variable induced by the perfect. Although a full formalization of aspect in FID must be left for further research, we thus note that the property of the log-FID operator to impose *de dicto* readings universally promises to derive Doron’s generalization.

**Table:**

(xvii)	a.	Antje had bought a dress.
	b.	pro-logFID- tns_i_^past^ λ_i_[Antje tns_i_^past^ perfect-t_i_ a dress]. This hypothesis may also derive the ban on *passé simple* in French FID, assuming as in Doron (1991) that the *passé simple* implies that the event happened before the reference time and the reference time overlaps with the utterance time. Contradiction ensues if both the reference time and the utterance time correspond to the protagonist’s time (see fn. 13).

34 This competition is reminiscent of Anvari’s (2019) ban on illeism precluding the use of third person (vs. first or second person) pronouns for speakers and addressees. Anvari investigates this ban in DD and ID (with indexical shift). In FID in languages without indexical shift, a first person pronoun, as we saw, is not available to refer to the logophoric protagonist in third person narrations. I will argue in Sect. 2.3 that this derives from the (independently motivated) hypothesis that the logophoric operator does not quantify over contexts. That said, I will also argue that improper contexts can be used in some (pragmatically appropriate) cases of FID (see Sect. 2.3), which seems to open the possibility that first person pronouns in FID could refer to a third person protagonist in those cases. As far as we know, this is however never the case, which further supports the hypothesis that *de se* bound pronouns prevail over free pronouns.

35 Charnavel (2019a, 2020) shows that in some cases, the same proposition can incorporate two different perspectives, as in (xviii), where *her own* and *strange* can be anchored to Paul’s daughter, and *horrible* and *herself* to his granddaughter.

**Table:**

(xviii)	[Paul’s daughter]_i_ explains that [_TP_ pro_i_ LOG [her_i_ own daughter]_k_’s strange diary relates [_DP_ pro_k_ LOG the media’s horrible remarks about herself_k_]. (Charnavel 2019a, p. 225) This does not question the logophoric homogeneity hypothesis, but derives from the hypothesis that a logophoric operator can be inserted within a clause under the appropriate semantico-pragmatic conditions. Here, the expressions *diary* and *relate* introduce Paul’s granddaughter’s perspective (expressed as the content of the diary). This is compatible with Charnavel’s hypothesis, under which a logophoric domain need not correspond to a full clause, but can be smaller; in fact, it must correspond to spell-out domains (basically TPs or other phrases with subject), which explains the behavior of apparently exempt anaphors. See further discussion about the consequences of this hypothesis for FID in Sect. 2.4.

36 As observed by Anand (2006) (cf. Deal 2020), indexical shift in ID respects an implicational hierarchy according to which if first and second person pronouns shift in a language, then adverbial indexicals do too (while the reverse does not hold—though see Schlenker 2003). According to them, this justifies positing operators that only shift the speaker and addressee coordinates, but not operators that only shift time and location coordinates. This is probably why Sharvit cannot simply assume that the FID operator shifts the time and location coordinates, but needs a hybrid account relying on both a shifting operator and a modification of the lexical entries of indexicals requiring dual context dependency.

37 This raises the question why *demain* remains alternatively available in FID. I argued for the domain of person that *de se* binding prevails over coreference with the protagonist, entailing for the domain of time that the time variable (in, e.g., *le lendemain*, i.e., *the day after t*) must be *de nunc* bound by log-FID if it yields the same interpretation as a free alternative. I further argued that a third person *de se* bound pronoun prevails over an improper first person pronoun (see fn. 35). In the domain of time (or space), this should entail that *de nunc* bound anaphoric adverbials should prevail over improper indexical adverbials. This seemed to be the case before the 19th century in languages like French (see fn. 25), but not afterwards, where we saw that the improper use of indexical adverbials is a hallmark of FID. Nevertheless, there is a difference between our examples in the person and time domains. Person pronouns are directly an index (endowed with some featural presuppositions), while adverbials like *le lendemain* ‘the next day’ or *la veille* lit. the wake, i.e., ‘the day before’ and indexicals like *demain* ‘tomorrow’ or *hier* ‘yesterday’ include other (relational) information. I would like to suggest that this information is not provided in the same way in the anaphoric and indexical variants, which makes the competition between both forms inapplicable. For instance, the same lexical items are not used in *la veille* or *hier*, and definite articles occur in the anaphoric forms (e.g., *la** veille*, *le l**endemain*), making them more complex than the indexical forms (e.g., *hier*, *demain*), and thus not comparable. Note that this entails that in DD, it is not directly because of competition with *demain* that *le lendemain* can’t be used; we should instead assume that the free time variable in *the lendemain* is specified as not denoting the actual time (cf. analyses of third person pronouns inducing a presupposition that their referent is not the speaker or addressee).

38 Although it is couched in a different framework, this hypothesis bears some resemblance with Schlenker’s (1999) hypothesis under which FID depends on matrix coordinates, which are not those of the actual context of utterance, but those of the fiction (see fn. 27). There is also some similarity with Maier’s (2017) attraction hypothesis (aiming to explain the use of pronouns in FID through unquotation; see Sect. 3.2), which relies on the idea that the story’s protagonists are more salient than the narrator. Schlenker (2004, p. 282), however, explicitly distinguishes his account from one relying on improper contexts, even if he accepts the existence of improper contexts (see fn. 49). Instead of positing an improper context in which the relation between the parameters is less strict than in Kaplan’s contexts, Schlenker distinguishes between a context of thought and a context of utterance (see Sect. 3.1.1). This makes available a double set of context coordinates. But interestingly, only a subset of those is actually accessible for indexicals in FID: speaker and addressee of the context of utterance, time and location of the context of thought, which is difficult to motivate (as reviewed in Sect. 3.1.1, Schlenker’s hypothesis based on presuppositions is problematic on several grounds; Eckardt simply stipulates it in lexical entries). The improper context hypothesis, on the contrary, only makes available the coordinates effectively used for indexicals.

39 I do not exclude the possibility that further factors (such as position and information structure in Anand & Toosarvandani 2019) may affect the specific behavior of *now*, *here* and/or *today*, but I must leave a full exploration of these indexicals for future research.

40 Interestingly, examples (97)-(98) involving shifted *tomorrow* in ID are also part of stories with no well-defined utterance context of narration.

41 This may suggest an explanation for why indexicals in FID are not found before the 19th century (at least in French), as mentioned in fn. 25: it may be the case that the narration before the 19th century did not meet the pragmatic conditions licensing the replacement of the actual context of utterance with an intended context of interpretation, i.e., the narrator is not appropriately remote from the context of interpretation.

42 I also leave for further exploration the distribution of adverbial indexicals in recursive FID (as I haven’t encountered examples of those). Can the time (and location) coordinate be resolved to different protagonists in those cases? In other words, can an improper context vary in the course of a narration?

43 Pearson (2015, p. 96) reports another possible interpretation according to which the discourse reports Kofi describing the dream to Mary. The same point holds under this interpretation. The difference is the type of attitude embedding the FID (dreaming or describing a dream, which seems to imply double embedding (*say* + *dream*)).

44 To decide between both types of hypotheses, it will be crucial to make progress on what the facts are exactly about faithfulness of the message in DD and FID and on how to capture them (in particular, we saw in Sect. 1.2.4 that there is in fact no verbatim requirement either in DD or in FID, but that DD seems to require more faithfulness than FID).

45 Under our log-FID account, we predict that proper names can refer to the protagonist only if they occur above the logophoric operator so as to avoid Condition C effects (see Charnavel 2019a, p. 227). This implies that log-FID is situated in vP rather than TP or CP in (149) (thus presumably precluding the use of an improper context here, see discussion in Sect. 2.4).

46 As noted by Schlenker (2005, p. 61), adverbial indexicals that can shift in ID such as French *dans deux jours* ‘in two days’ also raise a problem for Schlenker (2004). Given that it can shift in ID, *dans deux jours* must be treated as compatible with any variable of context; but in FID, it is not compatible with the narrator’s context of assertion. It must thus be assumed that the context of assertion can only be used in presuppositions associated with variables in FID.

47 In fact, Schlenker (2005, p. 62) notes that his system predicts that logophors such as *Konjunktiv I* cannot be relativized to the protagonist in FID (but can only be bound by an attitude verb occurring within FID).

48 Schlenker’s account of the historical present also raises problems regarding pronouns, which are predicted to depend on the (past) utterance context. For instance, the use of a first person pronoun in examples like (157) must in some cases imply the use of improper contexts (i.e., a context whose agent does not exist at the time of that context in the world of that context; see Schlenker 2004, p. 298). Worse, it predicts that first and second person pronouns should be able to refer to other individuals than the actual speaker or addressee. While Schlenker (2004, pp. 298-299) suggests that it is borne out for the first person pronoun, Schlenker (2005, p. 60) acknowledges that it is not for the second person pronoun.

49 In the case of logophoric *herself*, Maier could alternatively assume that *her* is unquoted while *self* is quoted, raising the problems of partial quotation mentioned later in the text; also crucially note that in principle, the whole reflexive *herself* should be unquotable under Maier’s account, which wrongly predicts the availability of reflexives bound *de re* as in (54).
